# Chromosome Synapsis Alleviates Mek1-Dependent Suppression of Meiotic DNA Repair

**DOI:** 10.1371/journal.pbio.1002369

**Published:** 2016-02-12

**Authors:** Vijayalakshmi V. Subramanian, Amy J. MacQueen, Gerben Vader, Miki Shinohara, Aurore Sanchez, Valérie Borde, Akira Shinohara, Andreas Hochwagen

**Affiliations:** 1 Department of Biology, New York University, New York, New York, United States of America; 2 Department of Molecular Biology and Biochemistry, Wesleyan University, Middletown, Connecticut, United States of America; 3 Institute for Protein Research, Osaka University, Suita, Osaka, Japan; 4 Institut Curie/Centre de Recherche, CNRS, UMR3664, Paris, France; National Cancer Institute, UNITED STATES

## Abstract

Faithful meiotic chromosome segregation and fertility require meiotic recombination between homologous chromosomes rather than the equally available sister chromatid, a bias that in *Saccharomyces cerevisiae* depends on the meiotic kinase, Mek1. Mek1 is thought to mediate repair template bias by specifically suppressing sister-directed repair. Instead, we found that when Mek1 persists on closely paired (synapsed) homologues, DNA repair is severely delayed, suggesting that Mek1 suppresses any proximal repair template. Accordingly, Mek1 is excluded from synapsed homologues in wild-type cells. Exclusion requires the AAA^+^-ATPase Pch2 and is directly coupled to synaptonemal complex assembly. Stage-specific depletion experiments further demonstrate that DNA repair in the context of synapsed homologues requires Rad54, a repair factor inhibited by Mek1. These data indicate that the sister template is distinguished from the homologue primarily by its closer proximity to inhibitory Mek1 activity. We propose that once pairing or synapsis juxtaposes homologues, exclusion of Mek1 is necessary to avoid suppression of all templates and accelerate repair progression.

## Abstract 2

Experiments in yeast indicate that one function of the synaptonemal complex is to disable chromosome-bound Mek1 kinase, thereby promoting DNA repair on fully paired meiotic chromosomes and helping to favor recombination between homologues over sister chromatids.

## Author Summary

Chromosome segregation errors during meiosis may cause infertility, fetal loss, or birth defects. To avoid meiotic chromosome segregation errors, recombination-mediated linkages are established between previously unattached homologous chromosomes. Such recombination events initiate with breaks in the DNA, but how these breaks are preferentially repaired using the distal homologous chromosome, rather than the physically more proximal sister chromatid of similar sequence, is not well understood. Meiotic repair-template bias in the budding yeast depends on the function of Mek1, a meiosis-specific protein kinase. Previous models suggested that Mek1 activity creates repair-template bias by suppressing repair with the sister chromatid. We found that Mek1 localizes on meiotic chromosomes until the homologues pair and closely align. Removal of Mek1 requires the assembly of a conserved zipper-like structure between meiotic chromosomes, known as the synaptonemal complex. DNA break repair is delayed in mutants in which Mek1 persists on closely aligned homologues. These findings suggest that persistent Mek1 activity can suppress repair from all templates, and that one function of the synaptonemal complex is to remove this activity from chromosomes. Our findings build on previous models to propose that Mek1 activity creates a local zone of repair suppression that is normally avoided by the spatially distant homologous chromosome to promote repair-template bias.

## Introduction

Meiosis is a specialized cell division that produces haploid gametes from diploid progenitors and is essential for sexual reproduction. The reduction in ploidy is achieved by a unique chromosome division phase (meiosis I) that segregates homologous chromosomes (homologues). Errors in this process are a leading cause of infertility, miscarriages, and birth defects in humans [1]. Proper meiosis I chromosome segregation in most organisms requires that each homologue pair be linked by at least one crossover. Crossover formation occurs during the extended prophase preceding meiosis I and is promoted by the programmed induction of DNA double-strand breaks (DSBs). Resection of these breaks exposes single-stranded DNA tails that invade a donor template for repair. A subset of strand-invasion reactions subsequently matures to form double Holliday junctions, which are generally resolved as crossovers [2].

To promote linkages between homologues, meiotic DSB repair is strongly biased toward using the homologue rather than the physically more proximal sister chromatid [3,4]. Available evidence, stemming mostly from studies in the budding yeast *Saccharomyces cerevisiae*, suggests that homologue bias results primarily from suppression of repair from the sister template. In yeast, this barrier to sister repair is mediated to a large extent by the chromosomal kinase Mek1, a meiosis-specific orthologue of mammalian CHK2 kinase that is recruited to the axial element structures of meiotic chromosomes upon DSB formation [3,5]. Mek1 recruitment requires the phosphorylation of the chromosome axis protein Hop1 on threonine 318 (T318) by the checkpoint kinases Mec1 (ATR) and Tel1 (ATM) [6]. Chromosomal recruitment leads to the dimerization and activation of Mek1 [7]. Mek1, in turn, phosphorylates a variety of targets, including the repair factors Rad54 and Rdh54, as well as histone H3 [8,9]. Phosphorylation of Rad54 inhibits its interactions with the recombinase Rad51 and is thought to help suppress sister-targeted repair along with additional Mek1 targets that remain to be identified [8]. In addition, Rad51 is kept inactive by its meiosis-specific inhibitor Hed1, which further biases repair towards the homologue [10–12]. Current models suggest that a DNA “tentacle” formed by the assembly of Rad51 and the meiotic recombinase Dmc1 on one end of a DSB interprets the suppressive signal established by Mek1, leading to preferred repair engagement with the homologue [3,4,13–15].

For homologue bias to be established, the DSB repair machinery must be able to distinguish homologue and sister templates. A probable mechanism included implicitly or explicitly in many models of homologue bias is that the sister chromatid is identified either by spatial proximity and/or through the cohesive linkages resulting from DNA replication [3,4,13–15]. As a result, the sister is subject to Mek1-dependent repair suppression, whereas the generally spatially more distant or unlinked homologue is not. An extension of the spatial proximity model is that if the homologue were to be transported within the range of Mek1 activity, it would also become suppressed as a template. Indeed, experimental hyperactivation of Mek1 also delays interhomologue repair [16], suggesting that Mek1-dependent repair suppression does not inherently distinguish between sister chromatids and homologous chromosomes.

The close alignment of homologous chromosomes is an essential part of meiotic prophase that in many organisms culminates in the assembly of the synaptonemal complex (SC). The SC is a conserved tripartite structure that assembles along the entire length of paired homologues during meiotic prophase [17]. In *S*. *cerevisiae*, SC assembly (synapsis) initiates at sites of crossover designation and centromeres [18–20] and involves the progressive deposition of Zip1, an extended coiled-coil protein that aligns homologous chromosomes at a fixed distance [21]. The function of the SC remains obscure. The SC is thought to stabilize pairing interactions, but homologues often remain co-aligned, albeit at a greater distance, in the absence of Zip1 [22]. Recent experiments have hinted at a signaling role for the SC. In several organisms, SC assembly is associated with a loss of chromosomal proteins, most notably yeast Hop1 and the orthologous HORMAD proteins in mouse [23–26], as well as the yeast DSB regulators Rec114 and Mei4, which require Hop1 for recruitment [27–29]. As DSB levels are elevated in SC mutants, these observations have led to the model that the SC acts as a feedback signal to suppress DSB formation on chromosomes that have engaged in crossover repair [10,30], although some evidence suggests that this suppression is not absolute [31,32]. Given that Mek1 recruitment also depends on chromosomal Hop1, the synapsis-associated loss of Hop1 would also be expected to affect Mek1 binding. Unexpectedly, however, Mek1 was reported to persist on synapsed chromosomes [33].

Here, we reinvestigated the chromosomal dynamics of Mek1 and its role in regulating meiotic DSB repair. We demonstrate that Mek1 is in fact eliminated from synapsing chromosomes and that removal requires Zip1-mediated recruitment of the AAA^+^-ATPase Pch2. Moreover, we show that DSB repair on synapsed chromosomes requires the function of Rad54, a target of Mek1-dependent inhibition. Importantly, failure to remove Mek1 from synapsed chromosomes leads to delays in DSB repair, indicating that Mek1 must be inactivated on fully engaged chromosomes to ensure timely completion of meiotic DSB repair.

## Results

### The SC Restricts Recruitment of Mek1

**Fig 1:**
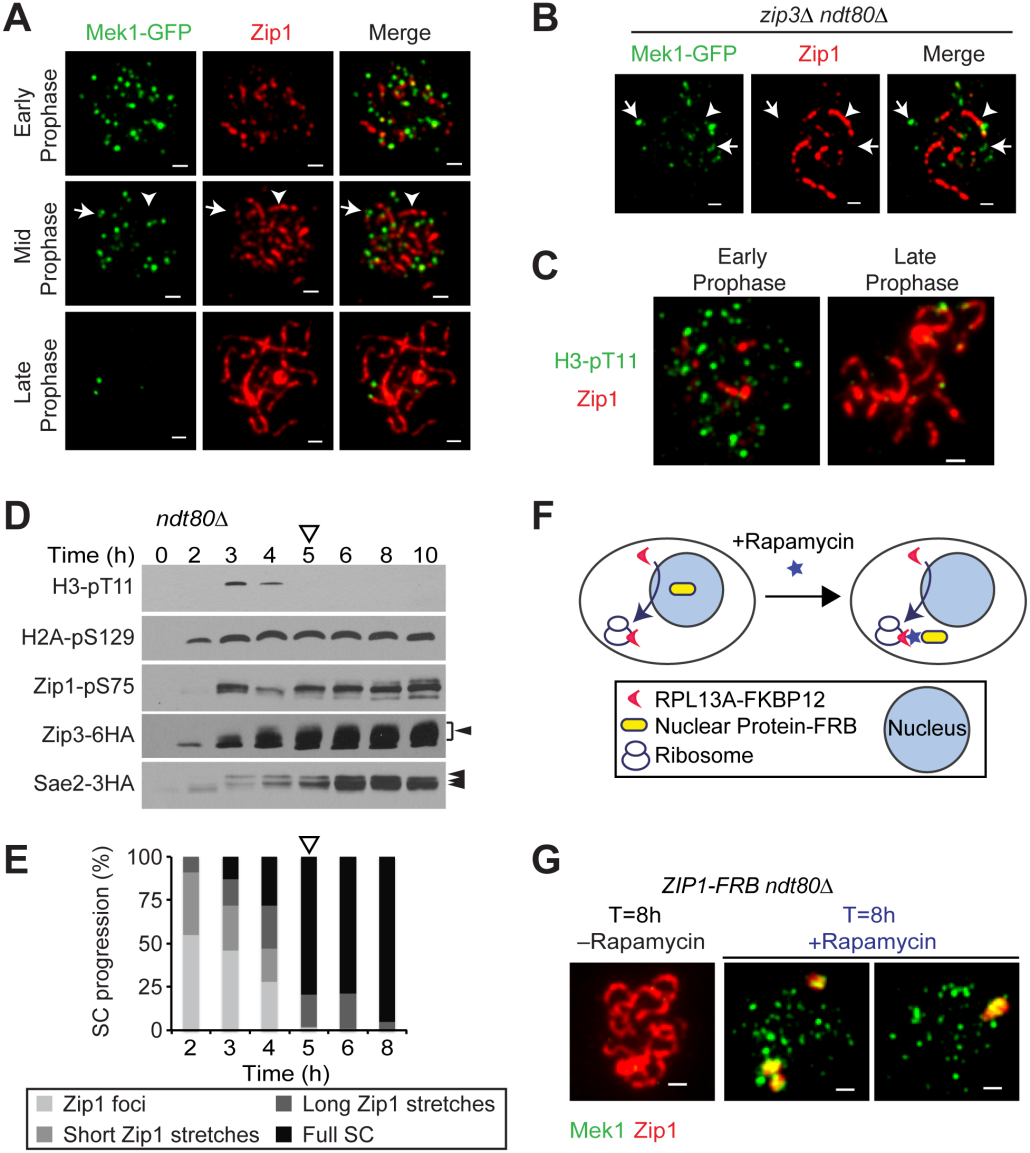
The SC restricts Mek1 localization on meiotic chromosomes. (A) Mek1-GFP fluorescence (green) and Zip1 immunofluorescence (red) on chromosome spreads from *MEK1-GFP ndt80Δ* (H7413) cells. Arrow marks Mek1-GFP fluorescence on an unsynapsed chromosomal region, arrowhead points to a representative chromosomal stretch associated with Zip1 but not Mek1. Scale bar is 1 μm. (B) Mek1-GFP fluorescence (green) and Zip1 immunofluorescence (red) on spread chromosomes of *zip3Δ ndt80Δ* (H7561) cells. Arrow points to Mek1-GFP fluorescence on an unsynapsed chromosomal region and arrowhead indicates Mek1 exclusion from a stretch of Zip1. (C) Immunofluorescence analysis of the Mek1 substrate histone H3-pT11 (green) and Zip1 (red) on chromosome spreads (H6179). (D) Western analyses of Mec1/Tel1 substrates from *ndt80Δ* (H6179, H8124, H7473) cells progressing synchronously through meiotic prophase. Arrows point to putative and documented phospho-shifts [6,35,36]. Open arrowhead marks the transition to inferred loss of Mek1 activity (based on the loss of H3-pT11 signal). Extracts for Zip3-6HA as well as Sae2-3HA westerns were made from heterozygous *ZIP3-6HA/+* (H8124) and homozygous *SAE2-3HA* (H7473) tagged strains, respectively. (E) Quantification of SC assembly progression at the indicated time points as determined by Zip1 immunofluorescence on spread nuclei (H6179). Open arrowhead indicates when majority of the nuclei have full SC. (F) Schematic for conditional nuclear depletion of proteins using the anchor-away technique (adapted from Haruki et al. Figure 1 [37]). (G) Immunofluorescence analysis of Mek1 (green) and Zip1 (red) on spread meiotic chromosomes of *ZIP1-FRB ndt80Δ* (H7421) cells. A meiotic culture of *ZIP1-FRB ndt80Δ* cells was split at T = 6 h and rapamycin was immediately added to one part of the culture for nuclear depletion of Zip1. Samples were collected from both cultures after 2 h for chromosome spreading. Scale bar is 1 μm.

To investigate the dynamics of Mek1 binding to meiotic chromosomes, we analyzed chromosome spreads using a functional Mek1-GFP construct. Nuclei were staged based on the progressive deposition of Zip1, marking the assembly of the SC along meiotic chromosomes. An *NDT80* deletion was used to prevent SC disassembly and Mek1 degradation due to exit from meiotic prophase [32,34]. Mek1 foci were abundant on chromosomes prior to the association of Zip1 (Fig 1A). However, in contrast to a previous report, which detected many apparent Mek1 foci on synapsed chromosomes [33], we observed a notable loss of chromosomal Mek1 from regions of extended Zip1 staining, such that Mek1-GFP foci were nearly undetectable when all chromosomes had assembled an SC (Fig 1A). The reason for this discrepancy is unclear but may be related to differences in strain background. The loss of chromosomal Mek1 signal was confirmed using a polyclonal antibody against Mek1 (S1A Fig), and was not due to a drop in Mek1 protein levels during meiotic prophase [34]. Examination of nuclei with partially assembled SC indicated that the disappearance of Mek1 foci was directly correlated with SC deposition even on individual chromosomes. As SC formation is relatively rapid in wild-type cells, we confirmed this observation in a *zip3Δ* mutant, in which chromosome synapsis is delayed and limited [18,20]. Like in the wild-type situation, Mek1-GFP signal was strongly reduced in synapsed regions but persisted on unsynapsed chromosomes in a *zip3Δ* mutant (Fig 1B). These data indicate that chromosome synapsis coincides with a loss of chromosomal Mek1.

The disappearance of Mek1 from chromosomes was mirrored by a loss of Mek1-dependent chromatin marks. Phosphorylation of histone H3 T11 requires Mek1 activity [9]. Immunostaining using an antibody specific for H3-pT11 revealed numerous foci on unsynapsed chromosomes but a near complete absence once chromosomes were synapsed (Fig 1C), implying that Mek1 is not active on synapsed chromosomes. To support this observation, we analyzed H3-pT11 by western blotting in a meiotic time course. Cells were blocked at the end of prophase using an *ndt80Δ* mutation to avoid secondary effects from Mek1 inactivation after prophase [38,39]. H3-pT11 first became detectable at 3 h after meiotic entry (Fig 1D), corresponding to the time of DSB induction. This timing correlated well with the phosphorylation of other meiotic checkpoint targets, including Zip1, Zip3 and Sae2. H2A-pS129 accumulated earlier presumably because of its role in premeiotic DNA replication [40]. Consistent with the analysis of chromosome spreads, H3-pT11 signal disappeared 5 h after meiotic induction, when most cells in the culture were completing SC formation (Fig 1E). The disappearance of H3-pT11 is in contrast to the other tested checkpoint targets, which remained phosphorylated during SC formation (Fig 1D and 1E).

Intriguingly, the SC-associated loss of Mek1 appeared to occur irrespective of persistent DNA repair intermediates. *zip3Δ* mutants are severely defective in DSB repair [18,41] and accumulate abundant repair foci marked by the Rad51 recombinase (S1B Fig). However, whereas Mek1 signal was largely restricted to unsynapsed regions in *zip3Δ* mutants, Rad51 foci were abundantly detectable on both unsynapsed and synapsed chromosomes. These data indicate that, at least in the absence of *ZIP3*, completed chromosomal DNA repair is not a prerequisite for the loss of Mek1 from synapsed chromosomes.

To test whether the SC is responsible for the loss of Mek1, we removed Zip1 from meiotic chromosomes. To circumvent potential pleiotropic effects of earlier roles of Zip1 in centromere pairing and DSB repair [42], we used the “anchor-away” technique [37] to conditionally deplete Zip1 from chromosomes that had already assembled SCs. In this technique, proteins tagged with the FRB domain of human mTOR are actively depleted from the nucleus after rapamycin addition due to interaction with a cytoplasmic anchor (a ribosomal protein fused to FKBP12; Fig 1F). Zip1 was quantitatively depleted from meiotic chromosomes within 2 h of rapamycin addition (S1C Fig). Nuclear depletion of Zip1-FRB throughout meiosis caused defects in sporulation and spore viability approximating the *zip1Δ* mutant, whereas untagged control strains treated with rapamycin retained wild-type spore viability (S1A and S1B Table). Strikingly, specific removal of Zip1 starting at the 6 h time point, when the vast majority of nuclei have fully synapsed chromosomes, caused rapid reaccumulation of Mek1 on chromosomes (17/20 nuclei; Fig 1G). We conclude that Zip1 assembly on chromosomes promotes the removal of Mek1 and is required to maintain Mek1 exclusion from synapsed chromosomes.

### Mek1 Loss Is Linked to the Chromosomal Elimination of the Hop1-pT318 Epitope

**Fig 2:**
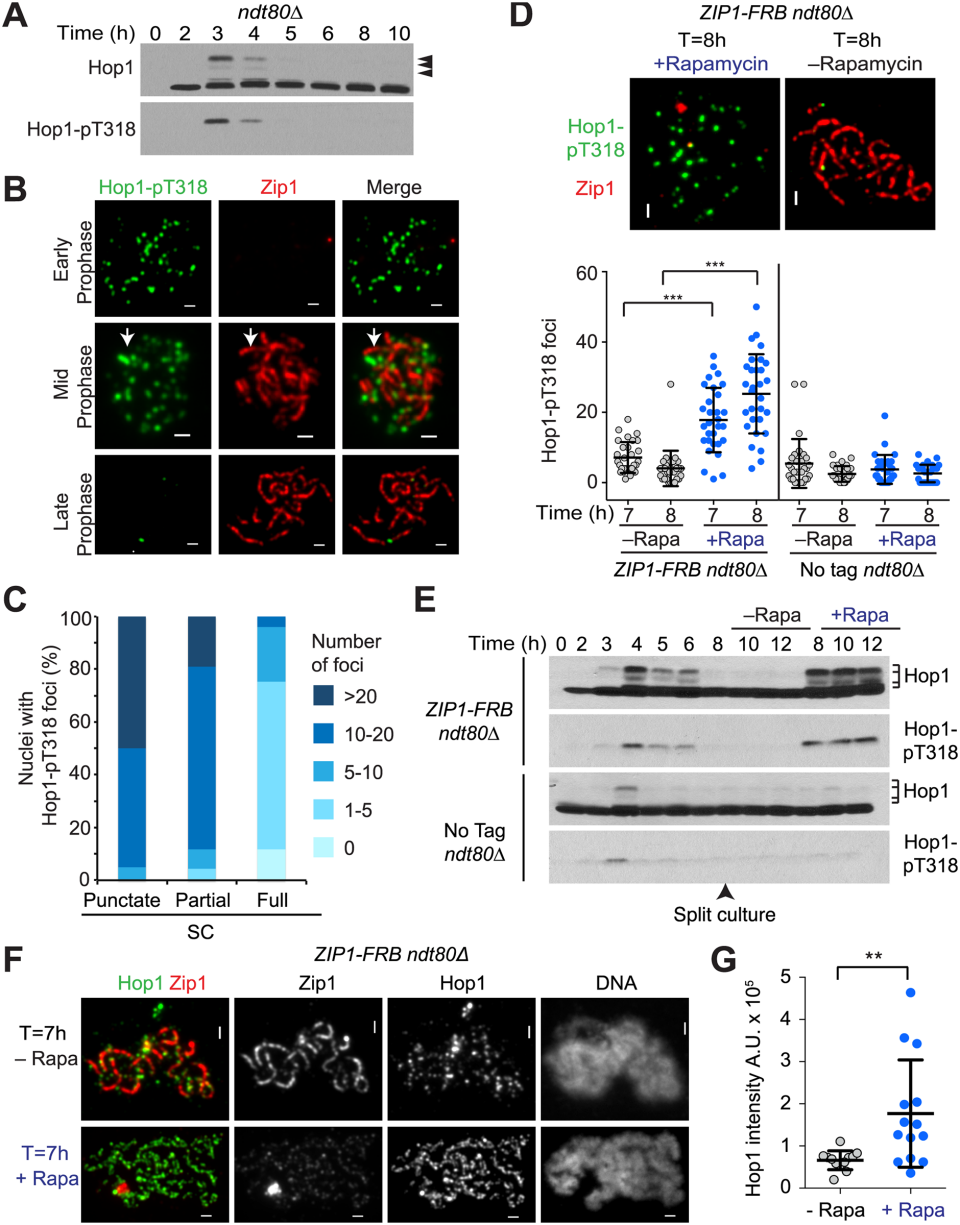
Loss of Mek1 from synapsed chromosomes correlates with a loss of the Hop1-pT318 epitope. (A) Western analysis of Hop1 and Hop1-pT318 from *ndt80Δ* (H6179) cells progressing synchronously through meiotic prophase. Arrows point to documented phospho-shifts of Hop1 [6]. (B) Immunofluorescence analysis of Hop1-pT318 (green) and Zip1 (red) on chromosome spreads (H6179). Arrowheads mark representative Zip1 stretches not associated with Hop1-pT318 signals. Scale bar is 1 μm. (C) Quantification of Hop1-pT318 foci on spread nuclei (H6179) at progressive stages of meiotic prophase as determined by SC morphology; *n* = 20, early prophase (punctate SC); *n* = 68, mid prophase (partial SC); *n* = 146, late prophase (full SC). (D-F) A culture of *ZIP1-FRB ndt80Δ* (H7421) and *ndt80Δ* control (H7137) cells was induced to undergo synchronous meiosis at T = 0 h and split at T = 6 h, after which rapamycin was added to one part of the culture for nuclear depletion of Zip1. Samples were collected at the indicated time points. (D) Upper panel: Immunofluorescence of Hop1-pT318 (green) and Zip1 (red) on spread chromosomes (H7421). Lower panel: quantification of Hop1-pT318 foci per spread nucleus (H7421, H7137). *n* = 30; error bars are standard deviation (S.D.) from the mean; *** *p* < 0.001, Mann-Whitney test. (E) Western analysis of Hop1 (upper panel) or Hop1-pT318 (lower panel) in protein samples from the indicated genotypes (H7421, H7137). (F) Immunofluorescence of Hop1 (green) and Zip1 (red) on spread chromosomes (H7421) at T = 7 h. Scale bar is 1 μm. (G) Quantification of total Hop1 intensity per spread nucleus for the experiment shown in (F). *n* = 13; error bars are S.D. from the mean; ** *p* = 0.005, Mann-Whitney test.

Chromosomal recruitment and activation of Mek1 requires the phosphorylation of Hop1-T318 [6], which may be subject to Zip1-dependent regulation. Consistent with this notion, the phosphorylation-dependent slower migrating forms of Hop1 disappear at the time of SC extension (Fig 2A) [40]. To more directly test the role of Hop1-T318, we raised a polyclonal antibody that specifically recognizes the phosphorylated form of this residue (Figs 2A and S2A). Immunofluorescence analysis revealed that, similar to Mek1, Hop1-pT318 foci were abundantly present in early prophase but disappeared coincident with SC assembly (Fig 2B and 2C). Moreover, we observed an increased accumulation of Hop1-pT318 signal in cell extracts and on meiotic chromosomes after Zip1 was depleted from the nucleus by anchor-away compared to the undepleted controls (Fig 2D and 2E). These data are consistent with a model whereby the disappearance of Mek1 from synapsed chromosomes is the result of a loss of Hop1-pT318 epitopes.

### Hop1-pT318 and Mek1 Persist on Synapsed Chromosomes When Pch2 Is Absent

**Fig 3:**
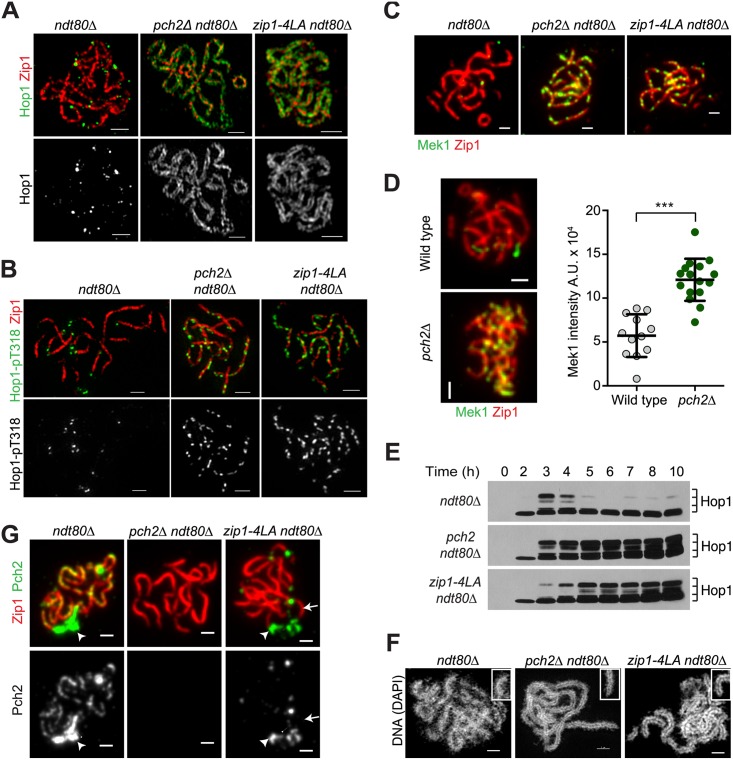
Zip1-dependent recruitment of Pch2 to synapsed chromosomes is required for removal of Hop1-pT318 and Mek1. Imaging and western analysis of wild-type cells, and *pch2Δ* and *zip1-4LA* mutants. All strains are also deleted for *NDT80* (*ndt80Δ* [H6179], *pch2Δ ndt80Δ* [H6639], *zip1-4LA ndt80Δ* [H6704]) except (D). (A and B) Structured illumination microscopy. Hop1 (green in top panels and single-channel in bottom panels) and Zip1 (red) are visualized in (A). Zip1 (red) immunofluorescence and Hop1-pT318 (green in top panels and single-channel in bottom panels) in (B). (C and D) Standard fluorescence microscopy. Mek1 immunofluorescence (green) and Zip1 immunofluorescence (red) in wild type, *pch2Δ* and *zip1-4LA* mutants at T = 6 h in (C). (D) Left panel: Mek1 immunofluorescence (green) and Zip1 immunofluorescence (red) in wild type *NDT80* cells (H574) and *pch2Δ NDT80* mutants (H3084). Right panel: Quantification of total Mek1 intensity per nuclear spread for the experiment. *n* = 12 for wild type and 16 for *pch2Δ*; error bars are S.D. from the mean; *p* < 0.001, Mann-Whitney test. (E) Western analysis of Hop1 in prophase-arrested *ndt80Δ*, *pch2Δ ndt80Δ* and *zip1-4LA ndt80Δ* mutants collected at the indicated time points. (F) Structured illumination microscopy of *ndt80Δ*, and *pch2Δ ndt80Δ* and *zip1-4LA ndt80Δ* mutants visualizing DAPI-stained DNA. Insets in (F) show individualized chromosome axes in *pch2Δ* and *zip1-4LA* mutants. (G) Standard immunofluorescence microscopy analysis of Pch2 (single-channel in the bottom panels and green in the top panels) and Zip1 (red) on spread meiotic chromosomes. Arrowhead points to the ribosomal DNA, which is enriched for Pch2 in *ndt80Δ* and *zip1-4LA ndt80Δ* strains but not in the *pch2Δ ndt80Δ* mutant. Arrow marks a Zip1 stretch that is not enriched for Pch2 in the *zip1-4LA ndt80Δ* mutant. Pearson’s coefficient of correlation for colocalization of Zip1 and Pch2 in *ndt80Δ* is (0.62 ± 0.09, *n* = 12) and in *zip1-4LA ndt80Δ* is (0.10 ± 0.07, *n* = 9). Scale bar is 1 μm.

Loss of at least some Hop1-pT318 epitopes is likely a secondary consequence of the reduced binding of Hop1 to synapsed chromosomes (S2B Fig) [23,25]. Indeed, Hop1 re-accumulates on chromosomes after depletion of Zip1 (Fig 2F and 2G). Hop1 removal from synapsing chromosomes requires the SC-bound AAA^+^-ATPase Pch2 [24,43,44] and super-resolution microscopy of the SC in *pch2Δ* mutants revealed an over-enrichment of Hop1 in two parallel tracts along the length of the lateral elements (Fig 3A). This effect was specific for Hop1, as the staining patterns of other SC lateral and central element components with respect to Zip1 appeared similar in wild type and *pch2Δ* mutants (S3A–S3C Fig). Significantly, Hop1-pT318 and Mek1 foci were visible on fully synapsed chromosomes in *pch2Δ* mutants (Fig 3B and 3C), which are almost never seen in wild-type cells. Chromosomal accumulation of Mek1 occurred independently of the *ndt80Δ* arrest (Fig 3D). Furthermore, phosphorylated Hop1 was abundant in *pch2Δ* whole-cell extracts (Fig 3E). These data indicate that Pch2 is responsible for the loss of Hop1-pT318 and Mek1 from synapsing chromosomes.

The persistence of Hop1 on synapsed chromosomes in the *pch2Δ* mutant is associated with unusually distinct parallel tracts of DAPI-stained chromatin along the lengths of chromosomes, a conformation only occasionally observed in short stretches on synapsed wild-type chromosomes (Fig 3F). Previous analyses had shown that *PCH2* is required for establishing separate domains of Zip1 and Hop1 along chromosomes, which fail to be formed in *pch2Δ* mutants [43,44]. We speculate that the distinctive parallel organization of chromosomes observed in *pch2Δ* mutants is another reflection of this altered chromosome structure, although we currently do not know whether the chromosomal persistence of Hop1 or Mek1 is responsible for this chromosome conformation in *pch2Δ* mutants.

### A Non-null Mutation of *ZIP1* Uncouples Chromosome Synapsis and Removal of Mek1

Our data suggest that removal of Mek1 depends on a Pch2-associated function during synapsis. Further analysis identified a non-null allele of *ZIP1* that assembles SC but fails to recruit Pch2 to synapsed chromosomes. Cells lacking a leucine-zipper in the coiled-coil region of Zip1 (*zip1-4LA*) [45] exhibited overall wild-type SC structure but lost all SC-associated Pch2 staining (Figs 3G and S3D–S3F). By contrast, the nucleolar pool of Pch2, which is independent of *ZIP1* [43], persisted in these mutants. Consistent with the failure to recruit Pch2 to the SC, *zip1-4LA* mutants retained large amounts of Hop1 on synapsed chromosomes (Fig 3A) and accumulated high levels of phosphorylated Hop1 and Mek1 (Fig 3B, 3C and 3E). Furthermore, as seen upon loss of *PCH2*, nuclear spreads of *zip1-4LA* mutants exhibited distinctly parallel DAPI tracks (Fig 3F). We note that the Pch2-mediated checkpoint, which specifically involves the nucleolar pool of Pch2 [43], remains active in *zip1-4LA* mutants [45]. These observations suggest that the Zip1-mediated recruitment or stabilization of Pch2 couples SC assembly to the removal of Mek1.

### PP4 Contributes to the Loss of Chromosomal Hop1-pT318

**Fig 4:**
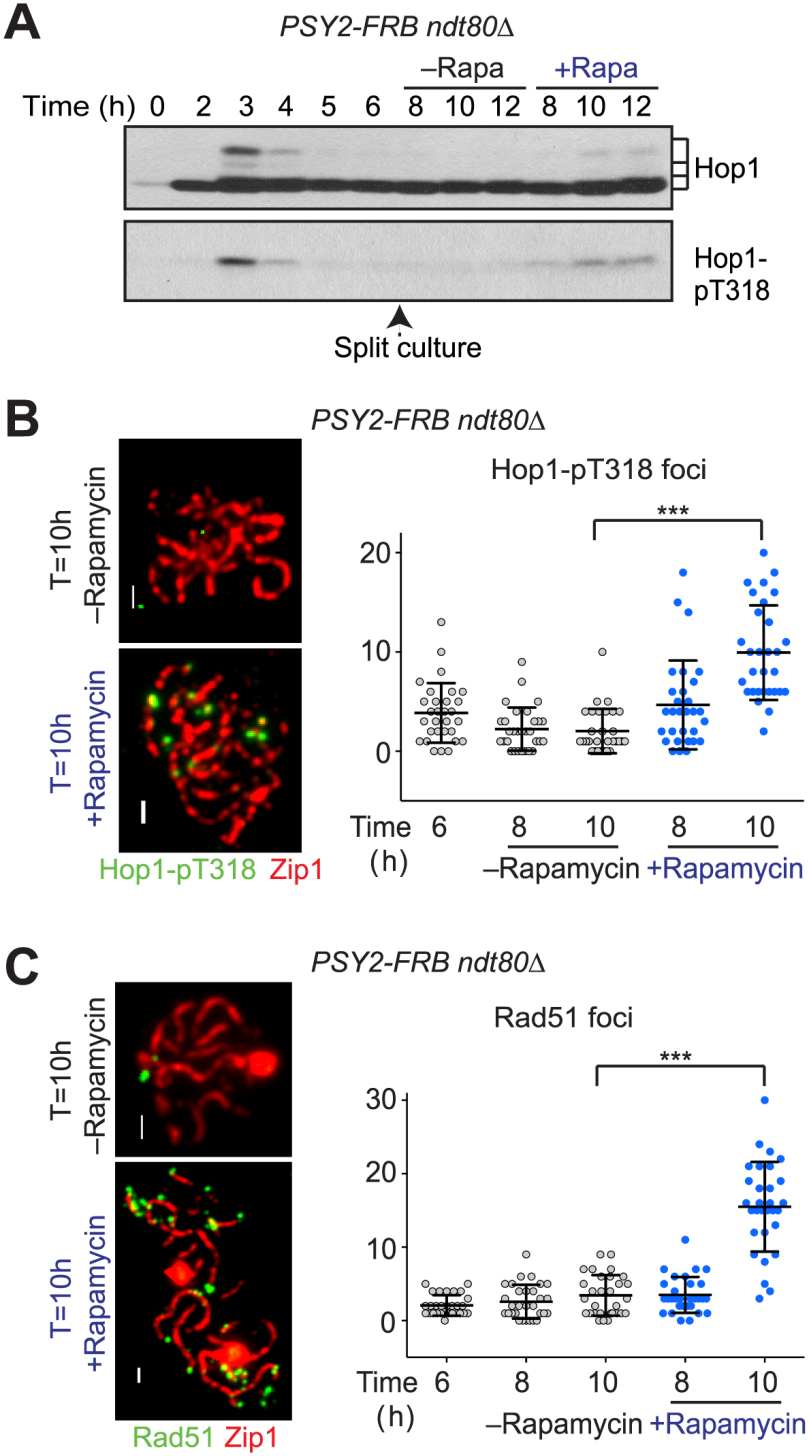
PP4 phosphatase contributes to removal of Hop1-pT318 and Mek1 from synapsed chromosomes. A culture of *PSY2-FRB ndt80Δ* (H7136) cells was induced to undergo synchronous meiosis at T = 0 h and split at T = 6 h, after which rapamycin was added to one part of the culture for nuclear depletion of Psy2-FRB. Samples were analyzed at the indicated time points. (A) Western analysis of Hop1 (upper panel) and phospho-Hop1 (lower panel) in the presence or absence of rapamycin. (B) Left panel: Immunofluorescence of Hop1-pT318 (green) and Zip1 (red) on spread meiotic chromosomes of *PSY2-FRB* cells in the presence or absence of rapamycin. Right panel: quantification of the number of Hop1-pT318 foci per spread nucleus. *n* = 30; error bars are S.D. from the mean; *** *p* < 0.001, Mann-Whitney test. (C) Left panel: Immunofluorescence of Rad51 (green) and Zip1 (red) on spread meiotic chromosomes of *PSY2-FRB* cells in the presence or absence of rapamycin. Right panel: quantification of the number of Rad51 foci per spread nucleus. *n* = 30; error bars are S.D. from the mean; *** *p* < 0.001, Mann-Whitney test.

We investigated whether the SC-associated loss of Hop1-pT318 is mediated by dephosphorylation in addition to Hop1 removal. PP4 protein phosphatase, comprising the catalytic subunit Pph3 and the cofactor Psy2, negatively regulates Hop1 phosphorylation [35,46]. To specifically interrogate the role of PP4 in Hop1 dephosphorylation during chromosome synapsis, we conditionally depleted the PP4-cofactor Psy2-FRB by anchor-away at the time of full synapsis. Nuclear depletion of Psy2 caused a modest accumulation of Hop1-pT318 signal in cell extracts (Fig 4A) and an increase in Hop1-pT318 focus number (Fig 4B), indicating that PP4 contributes to the removal of Hop1-pT318. Unlike in *pch2Δ* mutants, the increases in Hop1-pT318 signal were not associated with an increase in chromosomal Hop1 levels (S4A and S4B Fig). We do note, however, that the Hop1-pT318 signals often appeared at sites of discontinuity in Zip1 staining (Fig 4B), perhaps reflecting sites where Hop1 persists on synapsed chromosomes. These findings suggest that PP4 acts in parallel to Pch2 in eliminating Hop1-pT318 (and thus Mek1).

The reappearance of Hop1-pT318 foci upon PP4 depletion also presented a puzzle, as it implied an increasing number of unrepaired DSBs on synapsed chromosomes. DSB formation, a prerequisite for Hop1 phosphorylation and Mek1 recruitment [6,47], is thought to be largely shut down upon homologue engagement and synapsis [10,28,30,48,49], although several groups have reported continued presence of DSBs in *ndt80* mutants [30–32]. To test for the presence of unrepaired DSBs, we analyzed Rad51 focus number upon Psy2-FRB depletion. Nuclei with fully synapsed chromosomes displayed very few Rad51 foci when Psy2 was present (Fig 4C). By contrast, Psy2-FRB depletion led to a significant increase in Rad51 focus number on synapsed chromosomes that matched Hop1-pT318 accumulation (Fig 4C), suggesting an increased presence of DSBs. The accumulating Rad51 foci may reflect DSB repair intermediates that became destabilized upon PP4 depletion. Alternatively, they may represent continued DSB formation on synapsed chromosomes in the absence of PP4 activity. This latter possibility would imply that DSB formation continues on synapsed chromosomes.

### Zip1 Removal Reveals that DSBs Continue to Form on Post-synapsis Chromatin

**Fig 5:**
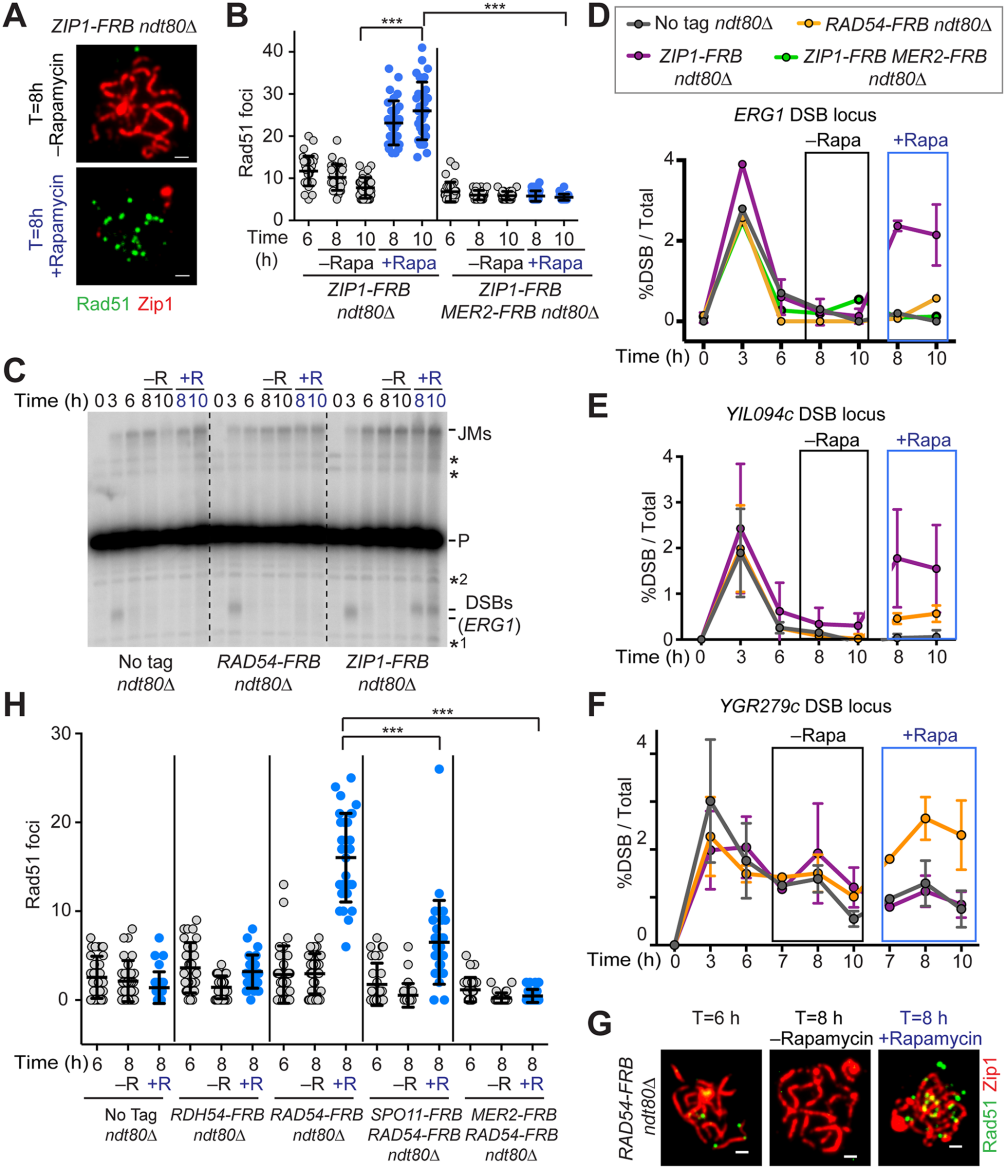
DSBs continue to form on synapsed chromosomes and require Rad54 for repair. Rapamycin was added to part of the culture at T = 6 h for depletion of various FRB-tagged nuclear proteins. Samples were analyzed at the indicated time points in the presence or absence of the drug. (A) Rad51 (green) and Zip1 (red) immunofluorescence on spread chromosomes of *ZIP1-FRB ndt80Δ* (H7421) cells. Scale bar, 1 μm. (B) Quantification of the number of Rad51 foci per spread nucleus upon depletion of Zip1-FRB (H7421) or co-depletion of Zip1-FRB and Mer2-FRB (H8359). *n* = 30; error bars are S.D. from the mean; *** *p* < 0.001 Mann-Whitney test. (C) Southern analysis to monitor DSBs at the *ERG1* locus in control cells (H7137) and before/after depletion of Rad54-FRB (H7121) or Zip1-FRB (H7421). P, parental unbroken fragment; JM, joint molecule repair intermediates; * nonspecific bands; *1 and *2 were used as anchors to measure the arbitrary molecular mass of DSB fragments in (S6C Fig). (D, E, F) Percentage of DSB fragments over total DNA at the *ERG1* (D), *YIL094c* (E) and *YGR279c* hotspot locus (F) for the indicated genotypes, time points and treatments. (G) Rad51 (green) and Zip1 (red) immunofluorescence on spread chromosomes of *RAD54-FRB ndt80Δ* (H7121) cells. Scale bar, 1 μm. (H) Quantification of the number of Rad51 foci per spread nucleus in control strains (H7137) or upon depletion of Rdh54-FRB (H7485), Rad54-FRB (H7121) or co-depletion of Rad54-FRB with Spo11-FRB (H7740) or Mer2-FRB (H7840). *n* = 30; error bars are S.D. from the mean; *** *p* < 0.001 Mann-Whitney test.

To begin to distinguish between these possibilities, we first asked whether DSB formation can be restored upon removal of the SC, which would indicate that DSB suppression associated with synapsis is reversible. We depleted Zip1-FRB by anchor-away and used immunofluorescence analysis of Rad51 to monitor DSB levels (Fig 5A). Zip1 depletion led to a significant increase in steady-state focus number of Rad51 (Fig 5A and 5B). This effect is not observed in untagged control cells (S5A Fig) and is mirrored by an increase in steady-state focus numbers of the single-stranded DNA-binding protein RPA (S5B Fig). Importantly, co-depletion of Zip1 and an essential DSB factor, Mer2, did not lead to an increase in Rad51 foci (Fig 5B). This outcome was not due to non-specific disruption of Mer2 by the FRB tag, because Mer2-FRB strains accumulated near wild-type levels of Rad51 foci prior to synapsis and produced fully viable spores in the absence of rapamycin (S5C Fig and S1A and S1B Table). These data indicate that new DSBs form in a Mer2-dependent manner after SC depletion.

We used physical assays at several endogenous DSB hotspots to monitor the occurrence of new DSBs upon depletion of Zip1 [30,50]. Electrophoretic separation of restriction-digested genomic DNA followed by Southern analysis allows detection of the larger unbroken DNA (parental size) as well as the faster migrating DSB fragments. DSB fragments reappeared at the *ERG1* hotspot in the *ZIP1-FRB* strain but not in the untagged control strain following rapamycin addition (Fig 5C and 5D). These DSBs were absent when Mer2 was co-depleted (Fig 5D and S6A Fig), indicating that they represent newly formed DSBs and are not the result of destabilized repair intermediates. DSB signal may be further increased due to the loss of Zip1 repair functions upon depletion [42]. A similar increase in DSBs was also observed at the *YIL094c* hotspot after Zip1 nuclear depletion (Fig 5E and S6B Fig). However, Zip1 nuclear depletion did not lead to significant DSB reappearance at the *YGR279c* or the *YCR047c* hotspot (Fig 5F and S6E Fig). Thus, although the increase in DSBs after Zip1 nuclear depletion is consistent with the notion that Zip1 prevents the formation of new DSBs on fully synapsed chromosomes [30], our data suggest that this suppression may occur in a locus-specific manner. We note that following Zip1 depletion, the DSB bands at several hotspots migrated at a higher molecular weight than DSB fragments observed in early prophase (Fig 5C and S6B–S6D Fig), suggesting that processing of DSB ends is altered in this situation.

### Ongoing DSB Formation on Synapsed Chromatin Is Rapidly Processed by Rad54

Given that depletion of PP4 led to an increase in Rad51 foci even in the presence of Zip1 and that previous studies have reported continued presence of DSBs in *ndt80* mutants [30–32], we asked whether some DSB formation is maintained when chromosomes appear fully synapsed in late prophase. To test this possibility, we depleted DSB repair factors from synapsed chromosomes to trap newly formed DSBs. We chose Rad54, which promotes Rad51-dependent DSB repair, and Rdh54, a Rad54-like protein that activates the meiosis-specific recombinase Dmc1. Dmc1 and Rdh54 are required for homologue-directed repair in meiosis [51,52], whereas Rad54 is inhibited by Mek1 to suppress intersister repair [8]. We reasoned since Mek1 is nearly absent on synapsed chromosomes, Rad54 may become active in this situation. We used anchor-away to deplete Rdh54-FRB and Rad54-FRB from synapsed chromosomes (Fig 5G and 5H). No increase in Rad51 focus number was observed upon removal of Rdh54 (Fig 5H), although nuclear depletion of Rdh54-FRB throughout meiosis caused an expected reduction in sporulation efficiency, indicating effective depletion (S1A Table). By contrast, Rad54 removal led to a strong increase in Rad51 focus number on synapsed chromosomes (Fig 5G and 5H). Although this finding may indicate that DSB formation continues on synapsed chromosomes, previous studies indicated that Rad51 also associates with undamaged DNA in the absence of Rad54 activity [53]. To address this possibility, we co-depleted a DSB-cofactor Mer2 or the DSB-inducing enzyme Spo11 with Rad54-FRB. Co-depletion of either factor significantly reduced Rad51 focus formation on synapsed chromosomes (Fig 5H). These data strongly suggest that DSB formation continues even when chromosomes appear fully synapsed, and that DSB turnover depends on Rad54.

Southern analysis indicated that DSB accumulation on synapsed chromosomes upon Rad54 depletion is locus-dependent. The accumulation of unrepaired DSBs was apparent at the *YGR279c* and *YCR047c* DSB hotspots (Fig 5F and S6D–S6F Fig), whereas the DSB signal at the *YIL094c* and *ERG1* hotspots did not increase substantially above background (Figs 5D, 5E and S6B). Interestingly, these patterns of DSB accumulation are opposite to the patterns observed upon Zip1 depletion (Figs 5D–5F and S6D–S6F). Thus, these differences may reflect the varying propensities of different genomic regions to synapse or differential dependence on *ZIP1* function for DSB repair. Alternatively, individual hotspots may differ in their dependence on Hop1/Mek1 for DSB formation and/or repair. In contrast to the slower-migrating DSB fragments after nuclear depletion of Zip1, the DSB fragments that appeared at the *YGR279c* and the *YCR047c* locus after nuclear depletion of Rad54 were faster migrating compared to DSBs in early prophase (S6D and S6F Fig; compare DSB pattern at T = 3 h to rapamycin-treated sample in Rad54-FRB). This migration pattern is consistent with hyperresection of DSBs ends and is typically observed when strand-invasion activity is blocked [54].

Despite the accumulation of Rad51 foci in Rad54-depleted nuclei, we observed no defect in SC structure (Fig 5G) and no increase in Hop1-pT318 focus number, overall Hop1 phosphorylation, or total chromosomal Hop1 signal upon Rad54 depletion (S7A–S7C Fig). This behavior is in stark contrast to the commensurate increase in Rad51 and Hop1-pT318 foci upon depletion of Zip1 (Figs 2D, 5A and 5B) or PP4 (Fig 4A–4C). These observations support the model that Zip1-dependent Hop1 removal and PP4 activity collaborate to prevent Hop1-T318 phosphorylation on synapsed chromosomes. We conclude that unrepaired DSBs do not lead to Mek1 recruitment when chromosomes appear fully synapsed.

### Mek1 Suppresses DSB Repair between Closely Engaged Chromosomes

**Fig 6:**
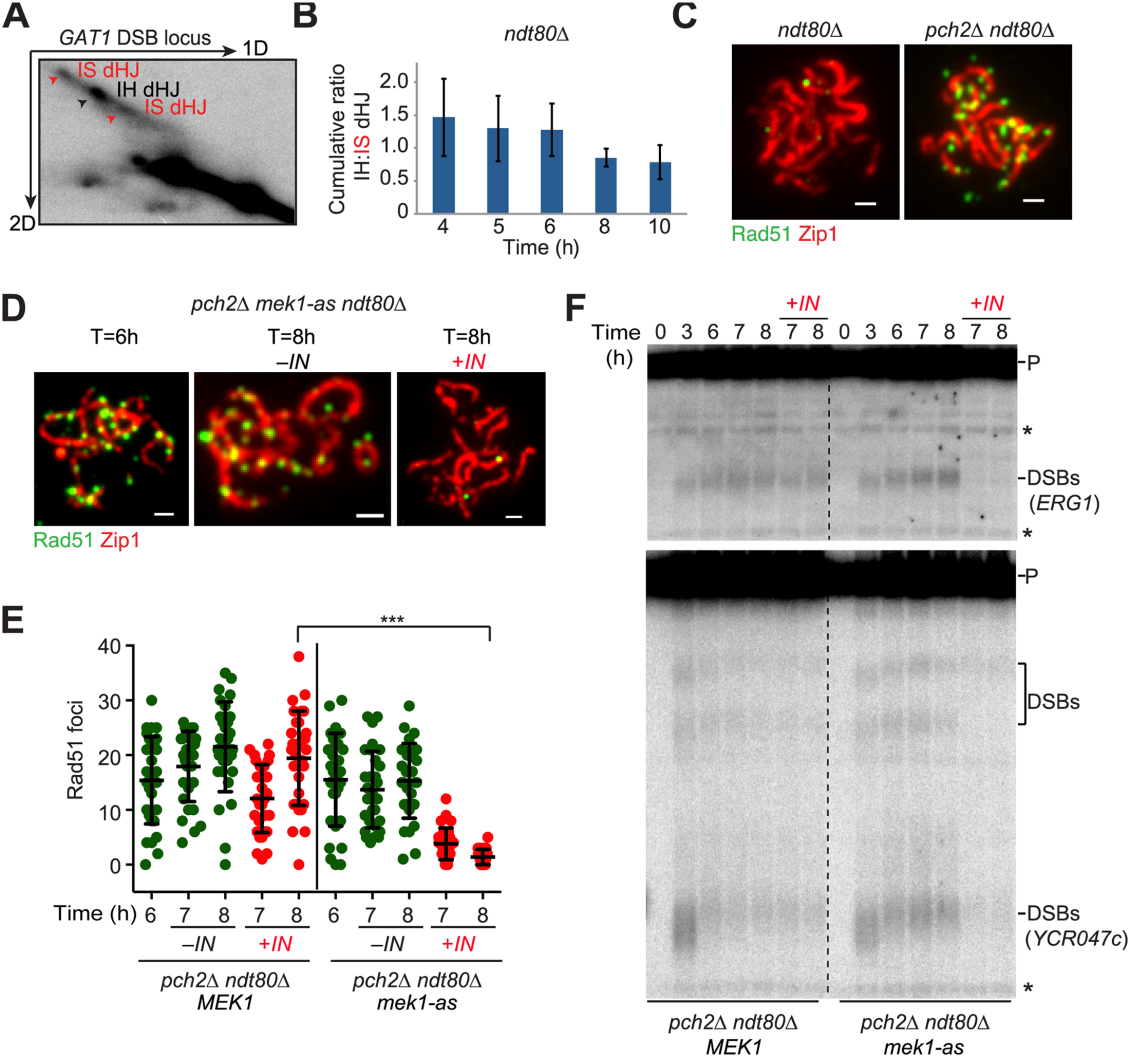
Mek1 activity on synapsed chromosomes suppresses DSB repair progression. (A) Snapshot of two-dimensional gel electrophoresis to resolve interhomologue (IH) and intersister (IS) dHJ species at the *GAT1* DSB locus at T = 4 h (H7036). (B) The IH:IS dHJ ratio at the *GAT1* DSB hotspot at progressive meiotic time points from two independent experiments. Error bars indicate signal range. (C) Immunofluorescence analysis of Rad51 (green) and Zip1 (red) on synapsed chromosomes in the presence (H6179) or absence of *PCH2* (H6639). Scale bar, 1 μm. (D-F) Cultures of *pch2Δ ndt80Δ* (H6639) and *pch2Δ ndt80Δ mek1-as* (H8360) cells were induced to undergo synchronous meiosis at T = 0 h and split at T = 6 h, after which the mek1-as inhibitor 1-NA-PP1 (*IN*) was added to one part of the culture to inactivate Mek1. Samples were analyzed at the indicated time points. (D) Rad51 (green) and Zip1 (red) immunofluorescence on synapsed spread chromosomes. Scale bar, 1 μm. (E) Number of Rad51 foci per spread nucleus of *pch2Δ ndt80Δ MEK1* and *pch2Δ ndt80Δ mek1-as* cells before and after inhibitor addition to inactivate mek1-as. *n* = 30; error bars are S.D. from the mean; *** *p* < 0.001 Mann-Whitney test. (F) Southern analysis to monitor DSBs at the *ERG1* (upper panel) or *YCR047c* (lower panel) locus. P, parental unbroken fragment; DSB, DSB sites at *ERG1* and at or near the *YCR047c* locus; * nonspecific bands.

The loss of Mek1 activity upon SC formation suggests that DSB repair on already synapsed chromosomes may not be constrained by homologue bias. To test this possibility, we investigated the formation of intersister (IS) and interhomologue (IH) double Holliday junction (dHJ) intermediates over time in *ndt80Δ* mutants at two DSB loci, *HIS4-LEU2* and *GAT1*. Engineered restriction site polymorphisms surrounding these DSB sites permit the separation of IS and IH repair intermediates by two-dimensional gel electrophoresis [30,55] (Fig 6A). As *ndt80Δ* mutants accumulate unresolved dHJs, analysis of IS and IH dHJs at a given time point will provide the cumulative average of template bias up until that time point. Analysis of the *HIS4-LEU2* hotspot revealed a strong IH bias that persisted over time (S8A Fig), consistent with previous results [56]. *GAT1* reproducibly exhibited a weaker IH bias (IH:IS ~1.5:1; Fig 6B and S8B Fig) than other strong DSB hotspots (IH:IS ~4:1) [10,56], but still substantially higher than the IH:IS ~1:9 template bias observed for mitotic DSB repair [57]. Notably, the cumulative IH:IS ratio at *GAT1* became progressively lower (Fig 6B and S8B Fig) consistent with decreased IH bias at later time points. These data support the notion that, at least at the *GAT1* locus, meiotic repair constraints are relaxed after chromosomes are fully synapsed. Because technical difficulties precluded us from analyzing IH bias at additional loci, we do not know to what extent this effect extends to other DSB hotspots.

**Fig 7:**
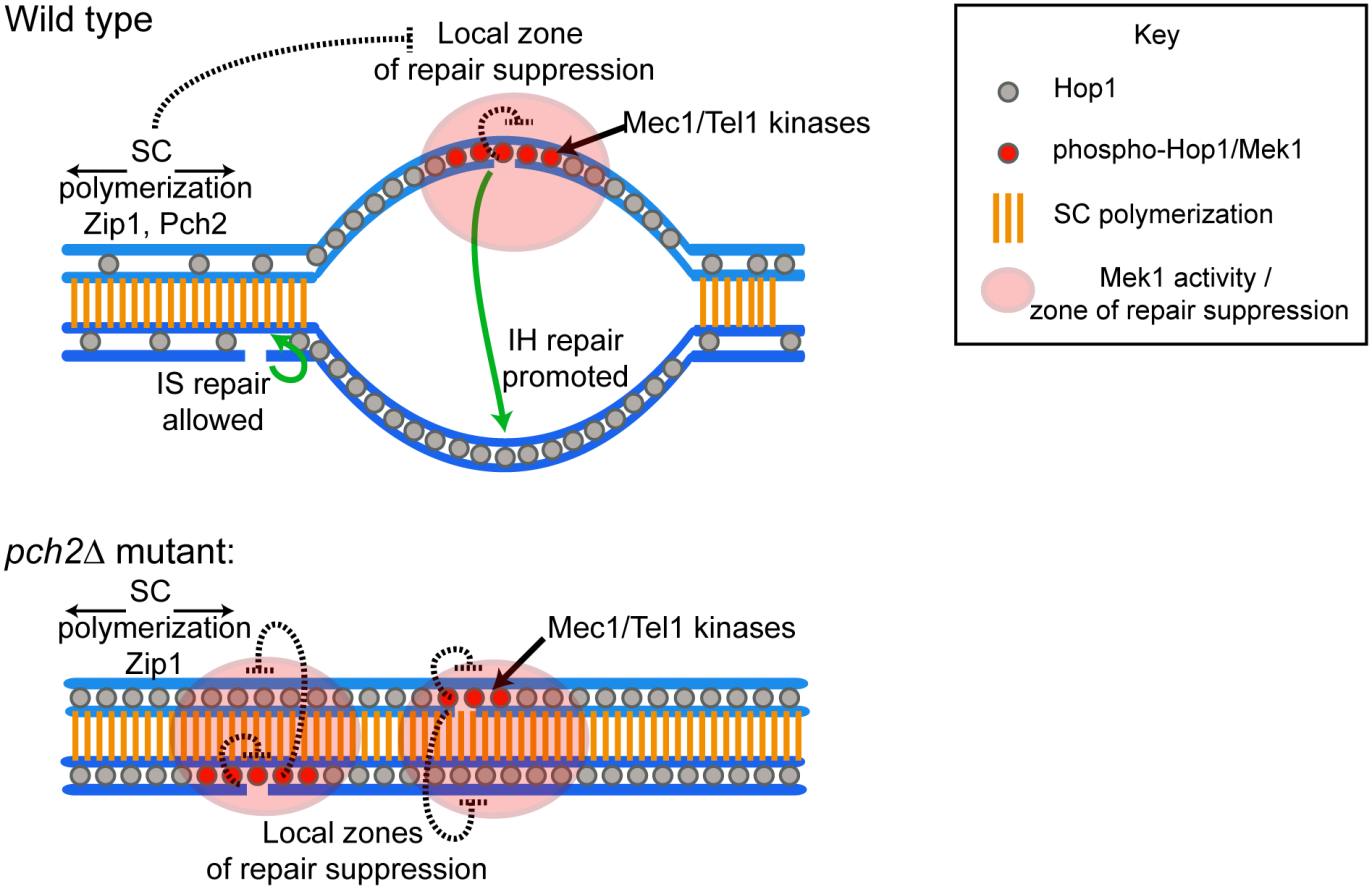
Model for synapsis-mediated elimination of Mek1-imposed repair constraints. Synapsis initiates at crossover-designated sites and centromeres (not depicted) and propagates along chromosomes to remove Hop1 and Mek1 in a chromosome-autonomous manner. This process alleviates local repair constraints imposed by Mek1 and switches repair from a strong homologue bias on unsynapsed chromosomes to an availability of both sister and homologue as repair templates on synapsed chromosomes. In a *pch2* mutant, chromosomal Mek1 activity persists and creates local repair constraints on all available templates, thereby causing a delay in DSB repair progression.

A major mechanism of establishing homologue bias is to make repair from the sister chromatid more difficult [13,58]. One conceptually simple way to achieve this goal is to establish a Mek1-dependent “zone” of repair suppression, such that spatially proximal sequences (i.e., the sister) cannot easily be used as repair templates [13]. If so, then removal of Mek1 may be necessary once chromosomes are aligned, as alignment would also place the homologue in this zone of repair suppression, thereby rendering repair from all templates equally difficult (see Fig 7). This model predicts that unrepaired DSBs should accumulate in cells that fail to remove Mek1 from synapsed chromosomes. Indeed, we observed an accumulation of Rad51 foci on fully synapsed chromosomes of *pch2Δ* mutants (Fig 6C). Rad51 accumulation occurred independently of the *ndt80Δ*-mediated prophase arrest (S8C Fig) and is consistent with previous observations showing a delay in DSB repair in these mutants [44,59]. To test whether the increased Rad51 foci on synapsed chromosomes are due to the persistence of Mek1 activity, we used an allele of Mek1 (*mek1-as*) that can be conditionally inactivated upon addition of a small molecule inhibitor (1-NA-PP1) [60]. Addition of the inhibitor after chromosomes were synapsed led to the disappearance of Rad51 foci in *pch2Δ mek1-as* mutants, whereas the foci persisted in untreated control cells (Fig 6D and 6E), suggesting rapid repair of DSBs once Mek1 was inactivated.

To confirm these results, we performed Southern analysis at the *ERG1* and *YCR047c* DSB hotspots. Consistent with the persistence of Rad51 foci, *pch2Δ* mutants accumulated DSBs at both hotspots (Fig 6F and S8D Fig). The persistent DSBs differed in their processing from DSBs formed in early prophase, similar to what was observed upon Zip1 depletion (S8E Fig). Importantly, the DSB bands were lost at both hotspots upon inactivation of Mek1 (Fig 6F and S8D Fig). Together, these results indicate that one function of the SC is to prevent Mek1 association with synapsed chromosomes in order to allow rapid DSB repair following homologue engagement.

## Discussion

Here, we used stage-specific depletion experiments to investigate the function of chromosome axis proteins and the SC in controlling meiotic DNA-repair signaling by Mek1 kinase. Our data suggest that Mek1 activity, while being essential for establishing meiotic repair template bias, creates a problem for DSB repair when all templates are in close proximity. We show that the SC transverse filament protein, Zip1, promotes Pch2-mediated exclusion of Mek1 from fully paired chromosomes. We further show that the ectopic presence of Mek1 on synapsed chromosomes prevents DSBs from undergoing Rad54-mediated turnover. Thus, by promoting the removal of Mek1, the assembly of Zip1 into SC structure can directly modulate DSB repair pathways. The chromosome-autonomous nature of SC assembly provides an obvious means to differentially control this process between chromosomes.

### A Model for Repair Template Selection

For repair template bias to be established, cells must be able to distinguish sister chromatids from homologous chromosomes. Our data point to a simple mechanism, whereby the primary determinant distinguishing sister from homologue is the spatial distance of the respective template from DSB-associated Mek1 activity (Fig 7). This model is in line with current models of template choice [3,4,13–15], which propose that a Mek1-dependent inhibitory domain suppresses repair progression from the proximal sister template, while the generally more distant homologue escapes this suppression. It is further supported by the observation that hyperactivation of Mek1 also delays interhomologue repair [16]. We argue, however, that a consequence of this simple setup is that once homologous chromosomes pair and establish close juxtaposition, Mek1 must be inactivated, so as not to place the homologue in the inhibitory domain and thus render all possible repair templates unfavorable. The stochastic nature of chromosome pairing would require this inactivation to be coupled to the behavior of individual chromosomes.

One prediction emerging from this model is that Mek1 activity along chromosomes must be spatially and temporally restricted, a notion supported by our experiments. In addition, Mek1 recruitment must be dynamic, as the genomic distribution of DSBs varies from cell to cell. Accordingly, Hop1 distribution is highly stereotyped and DSB-independent [27], whereas Mek1 recruitment is coupled to DSB induction [6,47].

The model that Mek1-dependent suppression of DSB repair is not inherently selective for the sister can also explain why DSBs persist in *pch2Δ* mutants. Chromosome pairing is unaffected in *pch2Δ* mutants [43], implying functional interhomologue repair interactions. However, repair completion may be suppressed because Mek1 activity persists on these chromosomes. This model may also explain why both crossover and non-crossover formation is delayed in *pch2Δ* mutants while the formation of single-end invasion intermediates occurs with wild-type kinetics [44]. Mek1 has been suggested to promote homologue bias in part by sequestering one DSB end in a quiescent state [14,61]. Perhaps persistent Mek1 activity hinders use of the sequestered end for completion of repair, thereby equally affecting crossover and non-crossover repair. Alternatively, the SC structure may create a situation that renders interhomologue repair structurally difficult, while Mek1 activity hinders repair with the sister template in *pch2Δ* mutants. Intriguingly, despite the severe repair delay, *pch2Δ* mutants display a wild-type level of spore viability [43,50]. This result implies that Mek1 suppression of DSB repair can be overcome with time and supports the notion that meiotic template choice is not absolute but rather the consequence of a kinetic barrier to repair [13].

We speculate that the presence of Mek1 may also contribute to the accumulation of Rad51 foci when PP4 or Zip1 are depleted from synapsed chromosomes (Figs 3C and 4A). In both experiments, Mek1 was bound to chromosomes that were allowed to fully pair prior to experimental manipulation (Zip1-FRB, Psy2-FRB), creating a situation similar to what is observed in *pch2Δ* mutants. Thus, although PP4 and Zip1 clearly have additional roles in recombination [35,42], the presence of Mek1 may further impair DSB turnover in these situations.

### An SC-Mediated Switch in Repair Constraints

The loss of Mek1 upon chromosome synapsis implies that meiotic repair constraints become progressively relaxed at late stages of meiotic prophase, such that repair perhaps transitions into a mitotic-like state. One likely consequence of this transition is that at least some of the DSB repair on synapsed chromosomes depends on the mitotic DSB repair factor Rad54, which presumably promotes Rad51-dependent repair. Moreover, as Rad54 activity mediates the disassembly of Rad51 filaments [62], it may also promote release of the second DSB end, which is thought to be held in a quiescent state by Rad51 [14]. The loss of Hop1 and Mek1 from synapsed chromosomes may also cause a down-regulation of Dmc1 activity, as Dmc1 no longer promotes meiotic DSB repair in the absence of either Hop1 or Mek1 [14]. Such loss of activity may explain why the DSBs that persist on synapsed chromosomes upon Rad54 depletion are not repaired and why depletion of the Dmc1-cofactor Rdh54 had little effect.

The successive implementation of repair constraints may be particularly important for the repair of DSBs in regions that lack an allelic sequence for repair, such as inversions or deletions, which, in fact, are repaired efficiently using the sister [13]. Chromosome synapsis is not dependent on sequence homology [63] and can thus spread into such regions from sites of SC nucleation. Conversely, constitutive binding of Zip1, as observed at yeast centromeres [64], may constitutively prevent the recruitment of Mek1 and thus activation of meiotic template bias. Indeed, deletion of Zip1 leads to an increase in interhomologue recombination specifically around centromeres in the absence of increased DSB formation [65], consistent with the model that centromeric DSBs are primarily repaired from the sister.

Our results complement a growing body of evidence that identifies the SC as a macromolecular signaling conduit. By extending out from sites of crossover designation, the SC may communicate successful engagement in crossover repair to the rest of the chromosome and trigger a profound switch in meiotic chromosome behavior, including the remodeling of meiotic chromosome structure and the dampening of further DSB activity [10,28,30,44,66]. Our work adds to this list the relaxation of meiotic repair constraints as a result of the SC-dependent removal of Mek1. Work in mice suggests that SC-dependent changes in chromosome structure and DSB activity are conserved [26,67]. It remains to be determined whether the same is true for the loss of repair constraints. Like in yeast, phosphorylation of HORMAD proteins is limited to unsynapsed chromosome axes in mice [68], and synapsis leads to strong TRIP13/Pch2-dependent depletion of chromosomal HORMAD proteins [26]. However, higher eukaryotes do not encode a clear Mek1 orthologue. Although CHK2 kinase could conceivably fulfill the role of Mek1 in these organisms, mouse CHK2 was recently shown to be required for checkpoint function without having a direct role in repair [69]. However, a role for the SC in regulating repair pathway choice is apparent in *Caenorhabditis elegans*, as partial depletion of the SC central region structure leads to increased interhomologue crossover events [70,71]. Although synapsis initiates independently of meiotic recombination in this organism [72], the change in repair parameters is associated with altered axial compaction [70], which may be functionally related to the altered DAPI patterns apparent in yeast *pch2Δ* mutants (Fig 3).

Ultimately, the transition in meiotic recombination mediated by the SC likely has at least two functions. First, it may preserve the pattern of crossover distribution by limiting the formation of additional crossovers [73]. Second, it minimizes the risk of aberrant repair events by restricting DSB numbers and by promoting the rapid repair of the DSBs that do form. Importantly, by executing this transition in *cis*, this feedback is robust to the inherently stochastic nature of chromosome pairing and meiotic crossover formation, and allows chromosomes to respond individually in a shared nuclear environment.

## Methods

### Ethics Statement

Antibody production was approved by the University Welfare Committee of New York University.

### Yeast Strains and Constructs

All yeast strains used are in the SK1 background except strains AM2981 and K303 (S3 Fig), which are in the BR1919-8B background. Genotypes are listed in S2 Table. Epitope tags and gene deletions were made by standard PCR-based transformations, except in the case of *ZIP1-FRB*. For construction of *ZIP1-FRB*, a previously published internally tagged *ZIP1-GFP*::*URA3* plasmid [74] was used and *GFP* replaced with the *FRB* sequence before integration at the *ZIP1* locus. *URA3* along with the wild-type *ZIP1* sequences was looped out on 5-FOA and a clone with a single copy of *ZIP1-FRB* was selected for further analysis.

### Synchronous Meiosis, Sporulation, Spore Viability

Cells were grown in liquid YPD culture at 23°C for 24 h and diluted at A_600_ 0.3 into presporulation media (BYTA; 50 mM sodium phthalate-buffered, 1% yeast extract, 2% tryptone and 1% acetate). The cells were grown in BYTA for 16 h at 30°C, washed twice in water and resuspended in sporulation media (0.3% potassium acetate) at A_600_ 2.0 to induce meiosis at 30°C. FACS analysis was used for all experiments to assay duplication of the genome and confirm synchronous meiotic initiation. Experiments to measure sporulation efficiency and spore viability were set up as synchronous meiosis as above and kept at 30°C in liquid sporulation media for 24 h.

### Conditional Nuclear Depletion or Inactivation

The anchor away technique was used to conditionally deplete proteins from the nucleus upon addition of rapamycin [37]. Rapamycin was added at a final concentration of 1 μM to the meiotic cultures at either meiotic induction (T = 0 h) or during pachynema (T = 6 h) except for depletion of Spo11-FRB or Mer2-FRB, where 2 μM rapamycin was added to the cells. *mek1-as1* [60] was conditionally inactivated by addition of the ATP analog, 1-NA-PP1 (Cayman Chemicals), at a final concentration of 2 μM, to the meiotic cultures during pachynema (T = 6 h).

### 1-D and 2-D Gel Electrophoresis, Southern Hybridization, and Quantitation

1-D gel analysis was performed as described in [75]; 2-D gel analysis of the dHJs was performed as described in [55]. Briefly, 15 mL samples were collected for the different time points and treated with 0.1% sodium azide. The cells were resuspended in 1 mg/mL Trioxsalen (Sigma) and the DNA was UV-crosslinked as described [35,76]. DNA was extracted and digested with the appropriate enzyme and then separated by two-dimensional gel electrophoresis. The DNA was transferred onto a ZetaProbe membrane (Biorad) by capillary transfer and detected by Southern hybridization. Probes for detection of dHJs at the *HIS4-LEU2* DSB locus are described [55]. A probe to assay the *YCR047c* locus is described [50]. Probes for *GAT1* and *ERG1* loci were amplified from genomic DNA with primers- 5′-caataagcaggtggagttgctgcg-3′, 5′-aaagatccaaagcccaccagattg-3′ and 5′-ggcagcaacatatctcaaggcc-3′ and 5′-tcaatgtagcctgagattgtggcg-3′ respectively. Primer pairs 5′ -attgtgcctgtaaccgaactgc-3′ and 5′ -agtggacgtagaaagaggagc-3′, 5′ -ttcctcgttcgtgacactactc-3′ and 5′ -tagctgccaaacccattctgc-3′ were used to generate the probes for *YIL094c* and *YGR279c* DSB hotspots, respectively. ^32^P-dCTP was incorporated into the probe using a Prime-It random labeling kit (Agilent). The Southern hybridization blot was exposed on a Fuji imaging screen and detected using a Typhoon FLA 9000 (GE). Hybridization signal was quantified using ImageJ software (http://imagej.nih.gov/ij/).

### Antibodies

Antibodies against phosphorylated Hop1 peptides (KLH-conjugated peptides: [H]- CKKLGNLLNS-pS-QASIQP -[NH2] and [H]- CKKQASIQP-pT-QFVSNNP -[NH2]) were raised in rabbits by Covance. The serum was affinity purified with the respective phospho-peptide, followed by adsorption against the unphosphorylated peptide using a SulfoLink Immobilization kit (Thermo Fisher Scientific). Affinity-purified pT318-Hop1 antibody was used at 1:100 for western analysis and 1:50 for immunofluorescence. The anti-Pch2 antibody was raised against the recombinant N-terminal 300 amino acids purified from *Escherichia coli*. An open-reading frame of the truncated Pch2 was PCR-amplified and inserted into the pET15b plasmid (Novagen), in which the N-terminus of the *PCH2* gene was tagged with 6x-Histidine. His-Pch2 protein was affinity-purified using a nickel resin as described by the manufacturers and used for immunization (MBL Co. Ltd). Rabbit anti-Hop1 antibody (kindly provided by N. Hollingsworth) was used at 1:10,000 for western analysis or 1:500 for immunofluorescence, rabbit anti-phospho-H3T11 (Millipore) and anti-Rfa2 antibodies (kindly provided by S. Brill) were used at 1:100 for immunofluorescence. Goat anti-Zip1 (Santa Cruz, SC-48716) was used at 1:200, goat anti-Zip1 (Santa Cruz, SC-15632) was used at 1:500, rabbit anti-Rad51 (Santa Cruz, SC-33626) was first pre-absorbed to *rad51Δ* meiotic spheroplasts and then used at 1:200, and rat anti-HA (Roche-11867431001) was used at 1:200. Secondary fluorescent-conjugated antibodies were obtained from Jackson Laboratory and were used for immunofluorescence after pre-absorption to yeast spheroplasts. HRP-conjugated secondary antibodies from Pierce were used for western analysis.

### Chromosome Spreads

Meiotic cells were collected at various time points, treated with 200 mM Tris pH7.5/20 mM DTT for 2 min at room temperature and then spheroplasted in 2% potassium acetate/ 1 M Sorbitol/ 0.13 μg/μL zymolyase T100 at 30°C. The spheroplasts were rinsed and resuspended in ice-cold 0.1 M MES pH6.4/ 1 mM EDTA/ 0.5 mM MgCl_2_/ 1 M Sorbitol. Two volumes of fixative (3% para-formaldehyde/ 3.4% sucrose) were added to the cells on a clean glass slide (soaked in ethanol and air-dried) followed by four volumes of 1% lipsol. The slide was tilted to mix the contents. Four additional volumes of the fixative were added to the slide and the samples were spread with a clean glass rod. After spreading was completed, slides were rinsed in 0.4% Photoflo (Kodak), dried overnight and stored at -80°C.

### Microscopy and Cytological Analysis

Images were collected on a Deltavision Elite imaging system (GE) equipped with an Olympus 100X lens/1.40 NA UPLSAPO PSF oil immersion lens and an InsightSSI Solid State Illumination module. Images were captured using an Evolve 512 EMCCD camera in the conventional mode and analyzed using softWoRx 5.0 software. Structured illumination microscopy was carried out on an OMX Blaze 3D-SIM super-resolution microscope equipped with a 6-line SSI Solid State Illumination module, 100X lens/1.40 NA UPLSAPO PSF oil immersion lens (Olympus) and three EVOLVE EMCCD cameras (housed at the Bio-imaging Resource Center, Rockefeller University). Super-resolution images for Fig 3 were collected on a Deltavision OMX V4 equipped with a 60X/1.42NA PLAPON oil immersion lens (Olympus). 100mW solid-state lasers were used along with three PCO sCMOS cameras for detection. Structured illumination reconstructions were carried out in softWoRx 6.1. Scatterplots were generated using the Graphpad program in Prism and statistical significance was assessed using a Mann-Whitney test.

## Supporting Information

S1 Data Numerical data underlying key figures in the article. (XLSX)

S1 Fig Loss of Mek1 localization correlates with SC assembly but not DNA repair. (A) Immunofluorescence analysis of Mek1 (green) and Zip1 (red) on chromosome spreads (H6179). Arrowheads mark representative chromosomal stretches associated with Zip1 but not Mek1. Scale bar is 1 μm. (B) Mek1-GFP fluorescence (green), Rad51 (white) and Zip1 immunofluorescence (red) on spread chromosomes of *ndt80Δ* (H7413) or *zip3Δ ndt80Δ* (H7561) cells. Arrow points to Mek1-GFP fluorescence on an unsynapsed chromosomal region and arrowhead indicates Mek1 exclusion from a stretch of Zip1. (C) Kinetics of Zip1-FRB depletion. Zip1-FRB was conditionally depleted from nuclei of *ZIP1-FRB ndt80Δ* (H7421) cells by addition of rapamycin to part of the culture at T = 6 h when the majority of meiocytes had fully assembled SC. Chromosome spreads were prepared from samples collected at the indicated time points and stained for Zip1. The SCs of 100 nuclei were classified for each time point.
(TIF)

S2 Fig Specificity of the Hop1-pT318 antibody and kinetics of Hop1 binding to chromosomes. (A) Western analysis of prophase extracts of the indicated genotypes using affinity-purified phospho-Hop1 antibody. The antibody does not recognize Hop1 protein in the *hop1(T318A*) mutant (H8210) or *HOP1* deletion (H3454) mutant but phospho-Hop1 bands were visible in the wild type (H6179). Nsp1 was used as loading control. (B) Immunofluorescence analysis of Hop1 (green) and Zip1 (red) on chromosome spreads at different stages of synapsis (H6179).
(TIF)

S3 Fig The relative arrangement of SC elements is unperturbed in *pch2Δ* and *zip1-4LA* mutants. Super-resolution microcopy of nuclear spreads of *pch2Δ ndt80Δ* (AM2981) in (A–C) and *zip1-4LA ndt80Δ* (K303) strains in (D–F) to visualize SC structure. Immunofluorescence of the SC central element protein Ecm11-MYC (red) and DNA staining (grey scale) is shown in relation to immunofluorescence of the SC lateral element protein Red1 (green) in (A) and (D), immunofluorescence of the C-terminus of Zip1 (green) in (B) and (E), and immunofluorescence of the N-terminus of Zip1 (green) in (C) and (F). Inset in the merged panels shows the relative position of the epitopes within the SC structure. Relative positions are also depicted in the schematic on left. Scale bar, 1 μm.
(TIF)

S4 Fig Hop1 binding to synapsed chromosomes is unaffected by depletion of PP4 (Psy2). A culture of *PSY2-FRB ndt80Δ* (H7136) cells was induced to undergo synchronous meiosis at T = 0 h and split at T = 6 h, after which rapamycin was added to one part of the culture for nuclear depletion of Psy2-FRB. Chromosome spreads were prepared after 4 h and the distribution of Hop1 (green) and Zip1 (red) in the presence or absence of rapamycin was analyzed by immunofluorescence in (A). (B) Total Hop1 immunofluorescence intensity per nuclear spread was quantified with or without rapamycin treatment.
(TIF)

S5 Fig DSB repair factors that are chromosome-associated in early prophase are reduced on synapsed chromosomes but increase upon Zip1 nuclear depletion. Rapamycin was added to part of a synchronous culture at T = 6 h (when most cells had fully synapsed chromosomes) for nuclear depletion of FRB-tagged proteins. (A–C) Spread chromosomes were analyzed by immunofluorescence for Rad51 or RPA, and foci were quantitated at the indicated time points in presence (+Rapa, blue circles) or absence of the drug (-Rapa, grey circles). (A) Rad51 foci per spread meiotic nuclei from *ZIP1-FRB ndt80Δ* (H7421) and an untagged *ndt80Δ* control strain (H7137). *n* = 30; error bars are S.D. with mean. (B) RPA (Rfa2) foci per spread meiotic nucleus from *ZIP1-FRB ndt80Δ* (H7421) and *RAD54-FRB ndt80Δ* (H7121). *n* = 30; error bars are S.D. with mean; *** *p* < 0.001. (C) Steady-state level of DSBs in early meiotic prophase in different FRB tagged strains without addition of rapamycin. The number of Rad51 foci per spread meiotic nuclei as marker of DSBs in early meiosis prior to complete synapsis (see T = 3 h and T = 4 h in Fig 1E). *n* = 30; error bars are S.D. with mean. Tagging of DSB factors (Spo11 (H7793), Mer2 (H7839)) or repair factors (Rad54 (H7121), Rdh54 (H7485) does not severely compromise DSB competence, sporulation, or spore viability (see also S1 and S2 Tables).
(TIF)

S6 Fig DSBs are reduced but not abolished on synapsed chromosomes and require Rad54 for repair. Rapamycin was added to part of a synchronous culture at T = 6 h (when most cells had fully synapsed chromosomes) for nuclear depletion of FRB-tagged Zip1 (H7421), Rad54 (H7121) or control (H7137). (A) Southern analysis to monitor DSBs at the *ERG1* locus. P, parental unbroken fragment; JM, joint molecule repair intermediates; * nonspecific bands. (B) Southern analysis to monitor DSBs at the *YIL094c* locus. P, parental unbroken fragment; * nonspecific bands; DSB, DSB sites at *YIL094c* locus. Note: slower migrating DSB bands after Zip1 nuclear depletion (T = 8+R, 10+R) compared to early prophase DSBs (T = 3). (C) Comparison of electrophoretic mobility of DSB fragments at the *ERG1* locus in Fig 5C. Dashed lines highlight the positions of the respective maxima. (D) Southern analysis to monitor DSBs at the *YCR047c* locus. P, parental unbroken fragment; DSB (*YCR047c*) or DSB, DSB sites at or near *YCR047c* locus. (E) Percentage of DSB fragments over total DNA at the *YCR047c* locus for the indicated genotype, time point and treatment. (F) Southern analysis to monitor DSBs at the *YGR279c* locus. P, parental unbroken fragment; * nonspecific bands; DSB, DSB sites at *YGR279c* locus.
(TIF)

S7 Fig Depletion of Rad54 from synapsed chromosomes does not trigger Hop1 phosphorylation. Rapamycin was added to part of a synchronous culture at T = 6 h (when most cells had fully synapsed chromosomes) for nuclear depletion of Rad54-FRB (H7121), Rdh54-FRB (H7485) or control cells (H7137). Samples were collected and analyzed at the indicated time points. (A) Number of Hop1-pT318 foci per spread nucleus with or without Rad54-FRB depletion. *n* = 30; error bars are S.D. from the mean. (B) Western analysis of Hop1 before and after depletion of Rad54-FRB or Rdh54-FRB. (C) Total Hop1 immunofluorescence intensity per nuclear spread was quantified with or without rapamycin treatment.
(TIF)

S8 Fig Mek1 activity suppresses DSB repair progression. (A) Ratio of interhomologue to intersister (IH:IS) dHJs at the *HIS4-LEU2* DSB locus over time in prophase-arrested *ndt80Δ* cells (H2640). (B) Two-dimensional gel electrophoresis to resolve interhomologue (IH) and intersister (IS) dHJ species at the *GAT1* DSB locus at different time points in meiosis (H7036). (C) Quantification of the number of Rad51 foci per spread nucleus in *NDT80* (H574) and *pch2Δ NDT80* (H3084) at T = 3 h. Only samples with complete SC were examined. *n* = 30; error bars are S.D. from the mean; *** *p* < 0.001 Mann-Whitney test. (D) Percentage of DSB fragments over total DNA at the *ERG1* locus for *pch2Δ ndt80Δ* (H6639) and *pch2Δ ndt80Δ mek1-as* (H8360) at the indicated time point and treatment. (E) Relative electrophoretic mobility of DSBs fragments at the *ERG1* locus in *pch2Δ ndt80Δ* (H6639) mutants (shown in Fig 6F). Dashed lines highlight the position of the respective maxima.
(TIF)

S1 Table Sporulation efficiencies of nuclear-depletion strains. (DOCX)

S2 Table Spore viabilities of nuclear-depletion strains. (DOCX)

S3 Table Strains used in this study. (DOCX)

## Abbreviations

dHJ: double Holliday junction
DSBs: double-strand breaks
IH: interhomologue
IS: intersister
SC: synaptonemal complex
T318: threonine 318
