# Supplementary figures and images for: Chromosome Synapsis Alleviates Mek1-Dependent Suppression of Meiotic DNA Repair
Source: PLoS Biol. 2016 Feb 12;14(2):e1002369. doi: 10.1371/journal.pbio.1002369 (PMC4752329; doi:10.1371/journal.pbio.1002369)

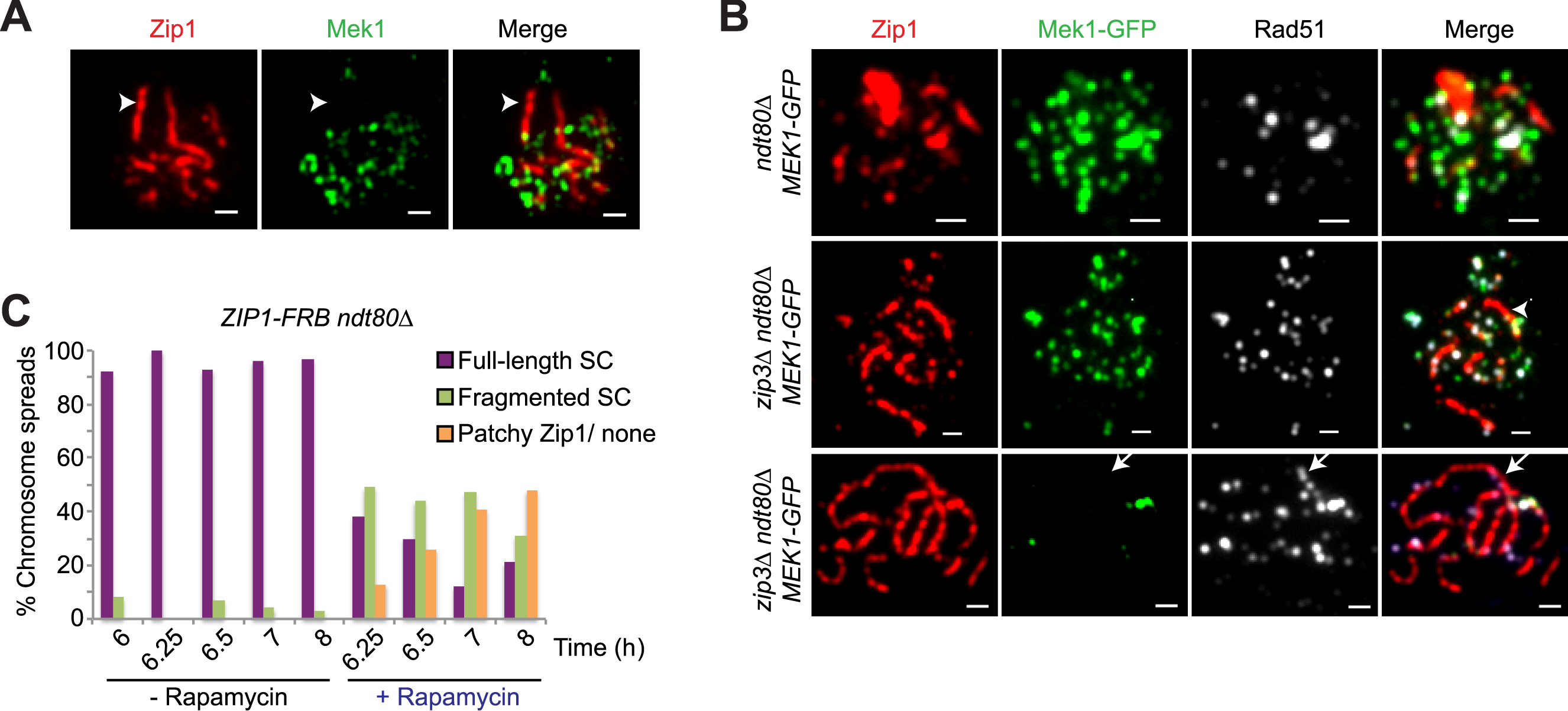

Supplement: S1 Fig — (A) Immunofluorescence analysis of Mek1 (green) and Zip1 (red) on chromosome spreads (H6179). Arrowheads mark representative chromosomal stretches associated with Zip1 but not Mek1. Scale bar is 1 μm. (B) Mek1-GFP fluorescence (green), Rad51 (white) and Zip1 immunofluorescence (red) on spread chromosomes of ndt80Δ (H7413) or zip3Δ ndt80Δ (H7561) cells. Arrow points to Mek1-GFP fluorescence on an unsynapsed chromosomal region and arrowhead indicates Mek1 exclusion from a stretch of Zip1. (C) Kinetics of Zip1-FRB depletion. Zip1-FRB was conditionally depleted from nuclei of ZIP1-FRB ndt80Δ (H7421) cells by addition of rapamycin to part of the culture at T = 6 h when the majority of meiocytes had fully assembled SC. Chromosome spreads were prepared from samples collected at the indicated time points and stained for Zip1. The SCs of 100 nuclei were classified for each time point. (TIF) [file pbio.1002369.s002.tif]

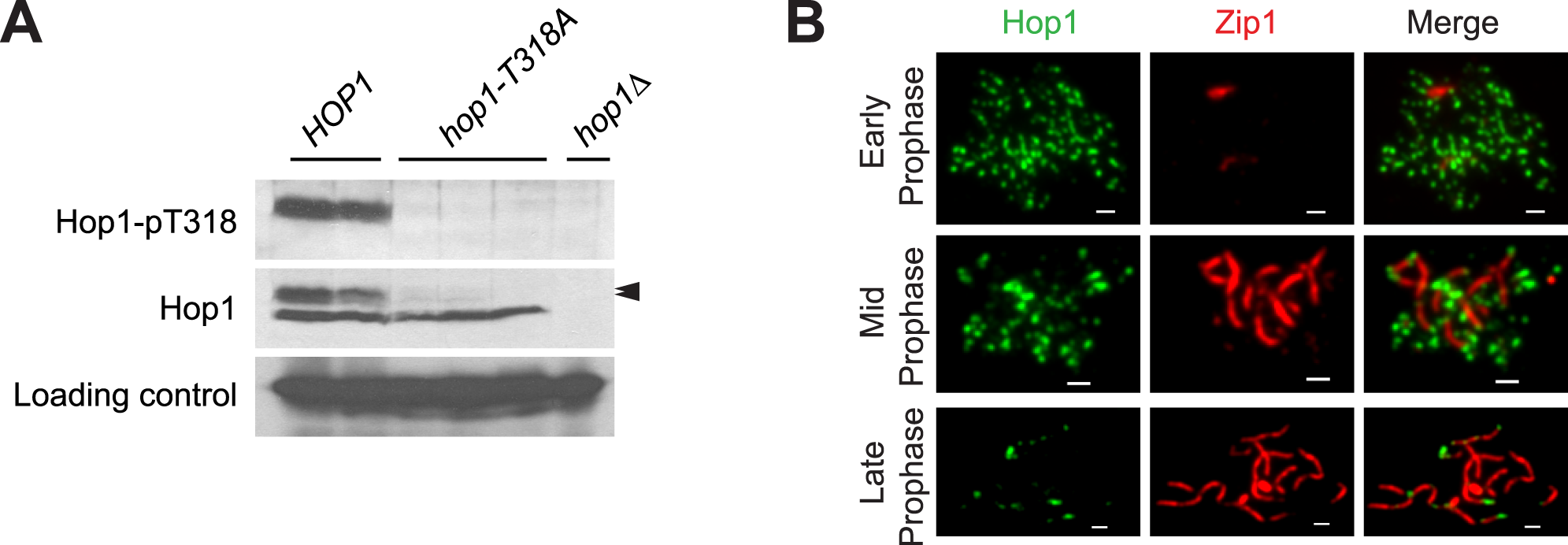

Supplement: S2 Fig — (A) Western analysis of prophase extracts of the indicated genotypes using affinity-purified phospho-Hop1 antibody. The antibody does not recognize Hop1 protein in the hop1(T318A) mutant (H8210) or HOP1 deletion (H3454) mutant but phospho-Hop1 bands were visible in the wild type (H6179). Nsp1 was used as loading control. (B) Immunofluorescence analysis of Hop1 (green) and Zip1 (red) on chromosome spreads at different stages of synapsis (H6179). (TIF) [file pbio.1002369.s003.tif]

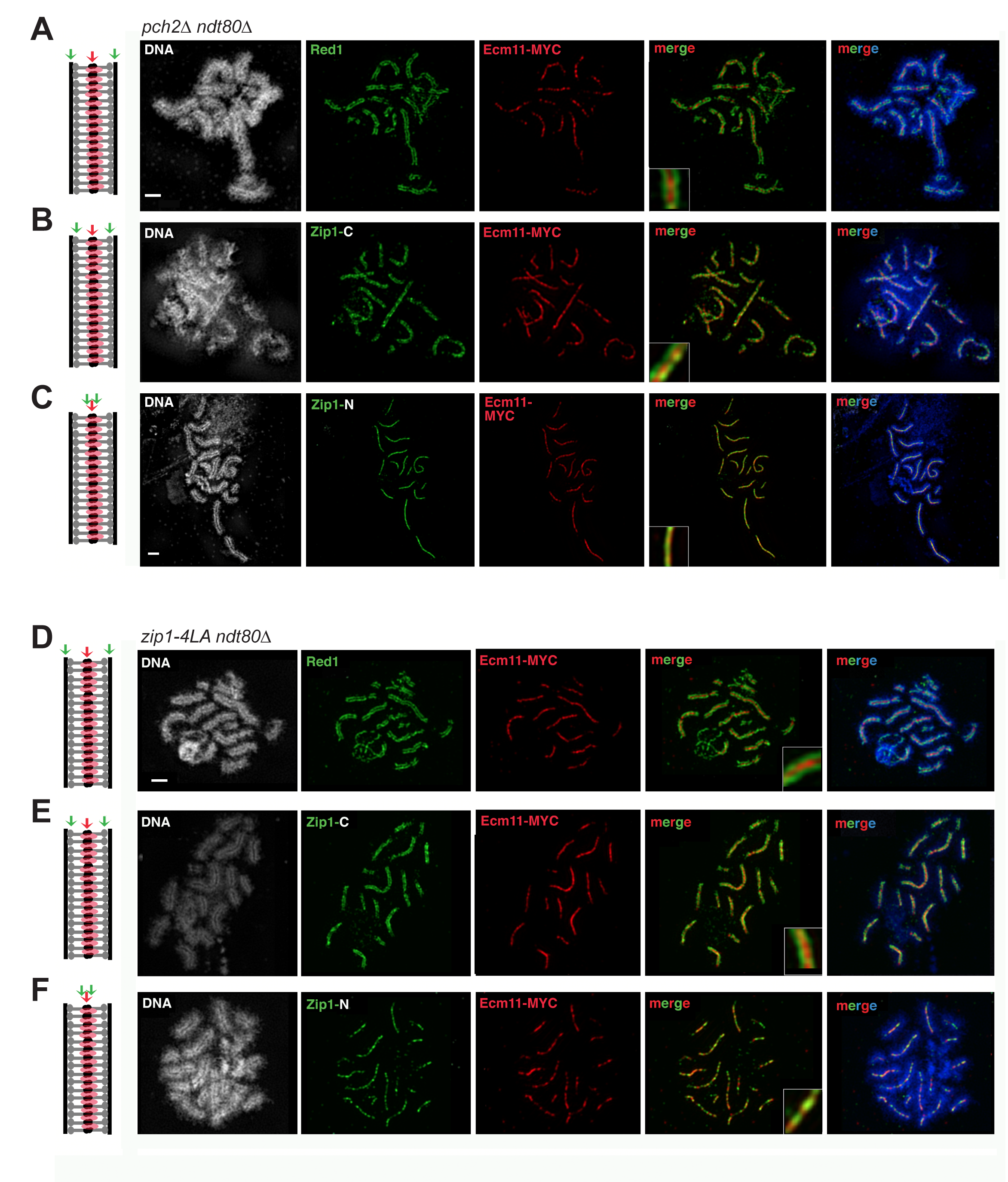

Supplement: S3 Fig — Super-resolution microcopy of nuclear spreads of pch2Δ ndt80Δ (AM2981) in (A–C) and zip1-4LA ndt80Δ (K303) strains in (D–F) to visualize SC structure. Immunofluorescence of the SC central element protein Ecm11-MYC (red) and DNA staining (grey scale) is shown in relation to immunofluorescence of the SC lateral element protein Red1 (green) in (A) and (D), immunofluorescence of the C-terminus of Zip1 (green) in (B) and (E), and immunofluorescence of the N-terminus of Zip1 (green) in (C) and (F). Inset in the merged panels shows the relative position of the epitopes within the SC structure. Relative positions are also depicted in the schematic on left. Scale bar, 1 μm. (TIF) [file pbio.1002369.s004.tif]

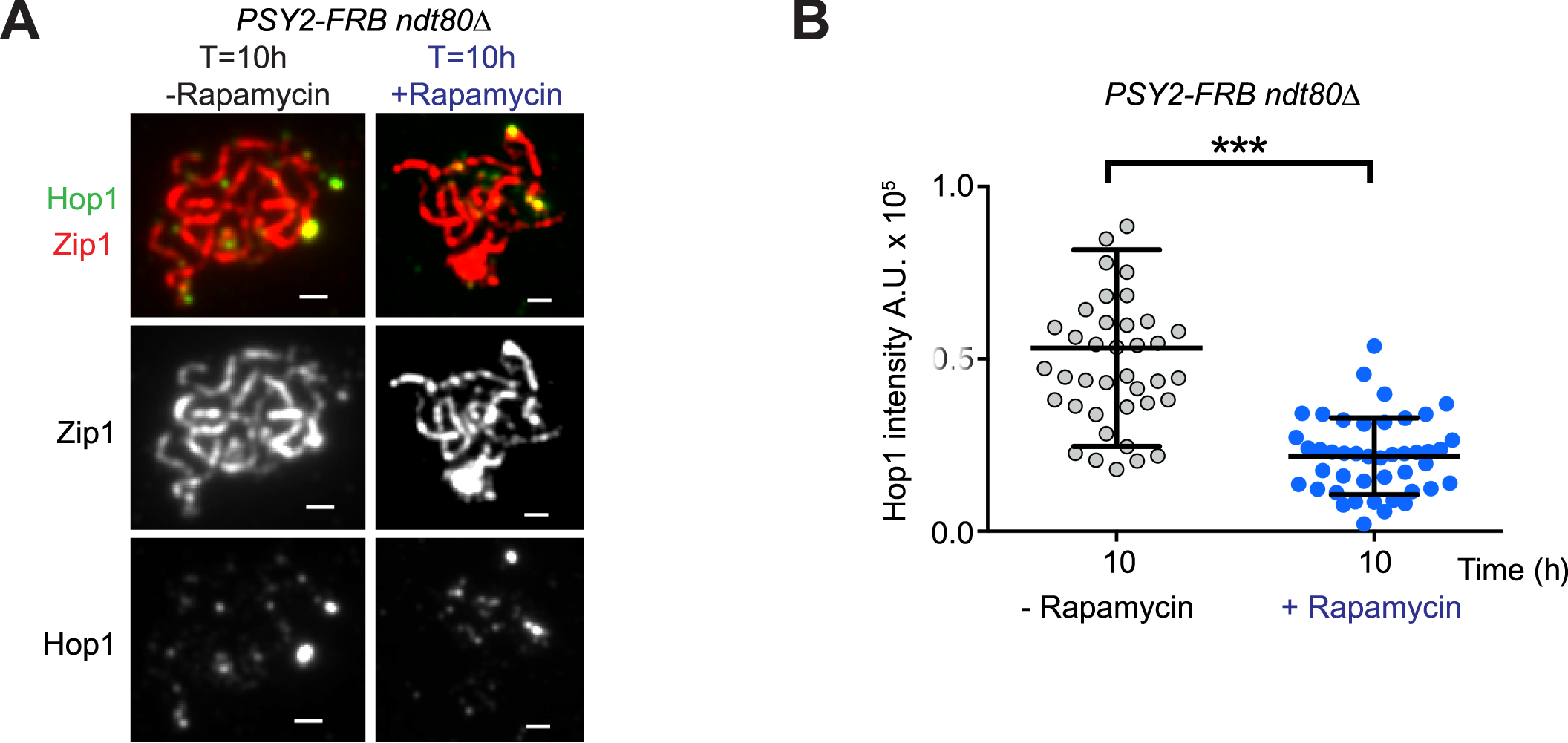

Supplement: S4 Fig — A culture of PSY2-FRB ndt80Δ (H7136) cells was induced to undergo synchronous meiosis at T = 0 h and split at T = 6 h, after which rapamycin was added to one part of the culture for nuclear depletion of Psy2-FRB. Chromosome spreads were prepared after 4 h and the distribution of Hop1 (green) and Zip1 (red) in the presence or absence of rapamycin was analyzed by immunofluorescence in (A). (B) Total Hop1 immunofluorescence intensity per nuclear spread was quantified with or without rapamycin treatment. (TIF) [file pbio.1002369.s005.tif]

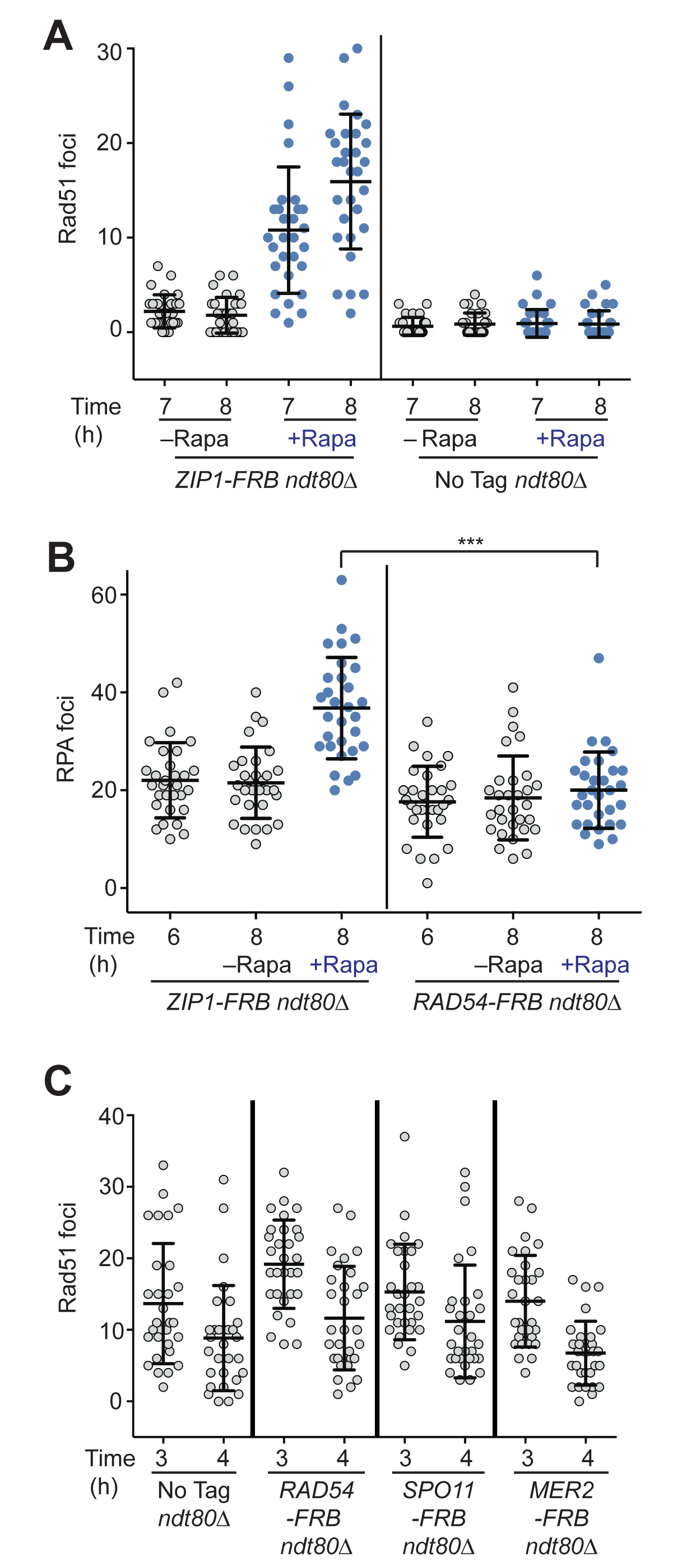

Supplement: S5 Fig — Rapamycin was added to part of a synchronous culture at T = 6 h (when most cells had fully synapsed chromosomes) for nuclear depletion of FRB-tagged proteins. (A–C) Spread chromosomes were analyzed by immunofluorescence for Rad51 or RPA, and foci were quantitated at the indicated time points in presence (+Rapa, blue circles) or absence of the drug (-Rapa, grey circles). (A) Rad51 foci per spread meiotic nuclei from ZIP1-FRB ndt80Δ (H7421) and an untagged ndt80Δ control strain (H7137). n = 30; error bars are S.D. with mean. (B) RPA (Rfa2) foci per spread meiotic nucleus from ZIP1-FRB ndt80Δ (H7421) and RAD54-FRB ndt80Δ (H7121). n = 30; error bars are S.D. with mean; *** p < 0.001. (C) Steady-state level of DSBs in early meiotic prophase in different FRB tagged strains without addition of rapamycin. The number of Rad51 foci per spread meiotic nuclei as marker of DSBs in early meiosis prior to complete synapsis (see T = 3 h and T = 4 h in Fig 1E). n = 30; error bars are S.D. with mean. Tagging of DSB factors (Spo11 (H7793), Mer2 (H7839)) or repair factors (Rad54 (H7121), Rdh54 (H7485) does not severely compromise DSB competence, sporulation, or spore viability (see also S1 and S2 Tables). (TIF) [file pbio.1002369.s006.tif]

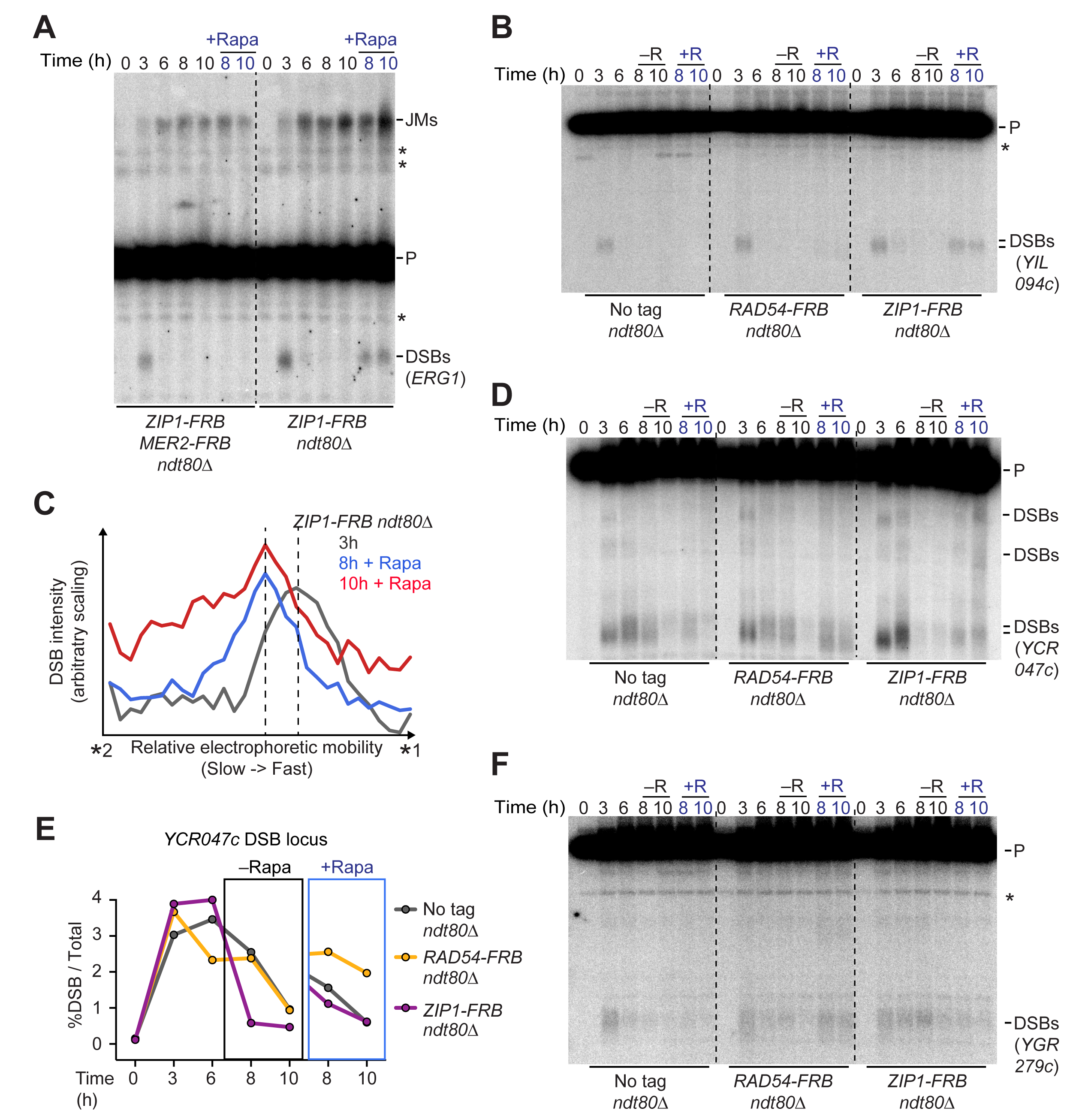

Supplement: S6 Fig — Rapamycin was added to part of a synchronous culture at T = 6 h (when most cells had fully synapsed chromosomes) for nuclear depletion of FRB-tagged Zip1 (H7421), Rad54 (H7121) or control (H7137). (A) Southern analysis to monitor DSBs at the ERG1 locus. P, parental unbroken fragment; JM, joint molecule repair intermediates; * nonspecific bands. (B) Southern analysis to monitor DSBs at the YIL094c locus. P, parental unbroken fragment; * nonspecific bands; DSB, DSB sites at YIL094c locus. Note: slower migrating DSB bands after Zip1 nuclear depletion (T = 8+R, 10+R) compared to early prophase DSBs (T = 3). (C) Comparison of electrophoretic mobility of DSB fragments at the ERG1 locus in Fig 5C. Dashed lines highlight the positions of the respective maxima. (D) Southern analysis to monitor DSBs at the YCR047c locus. P, parental unbroken fragment; DSB (YCR047c) or DSB, DSB sites at or near YCR047c locus. (E) Percentage of DSB fragments over total DNA at the YCR047c locus for the indicated genotype, time point and treatment. (F) Southern analysis to monitor DSBs at the YGR279c locus. P, parental unbroken fragment; * nonspecific bands; DSB, DSB sites at YGR279c locus. (TIF) [file pbio.1002369.s007.tif]

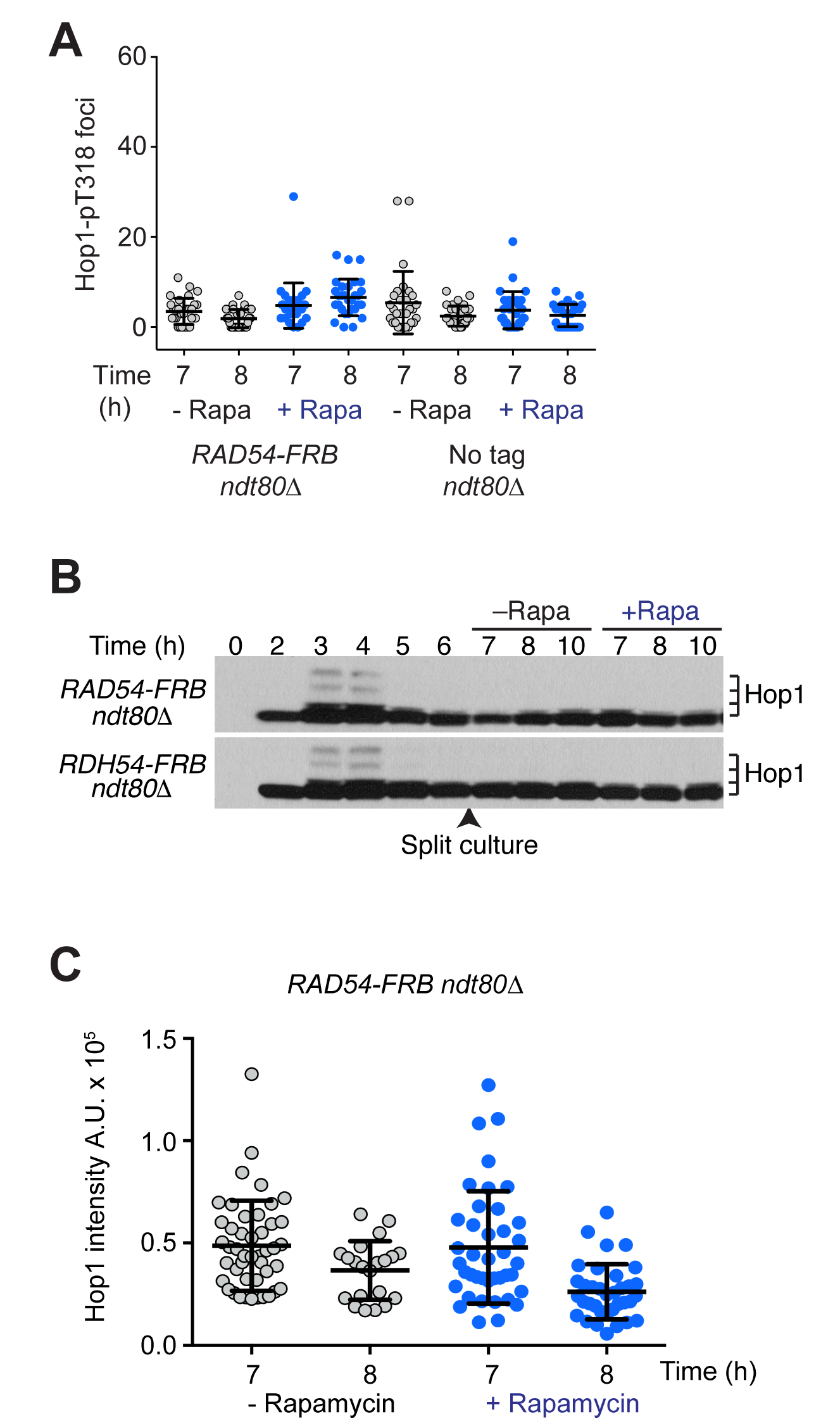

Supplement: S7 Fig — Rapamycin was added to part of a synchronous culture at T = 6 h (when most cells had fully synapsed chromosomes) for nuclear depletion of Rad54-FRB (H7121), Rdh54-FRB (H7485) or control cells (H7137). Samples were collected and analyzed at the indicated time points. (A) Number of Hop1-pT318 foci per spread nucleus with or without Rad54-FRB depletion. n = 30; error bars are S.D. from the mean. (B) Western analysis of Hop1 before and after depletion of Rad54-FRB or Rdh54-FRB. (C) Total Hop1 immunofluorescence intensity per nuclear spread was quantified with or without rapamycin treatment. (TIF) [file pbio.1002369.s008.tif]

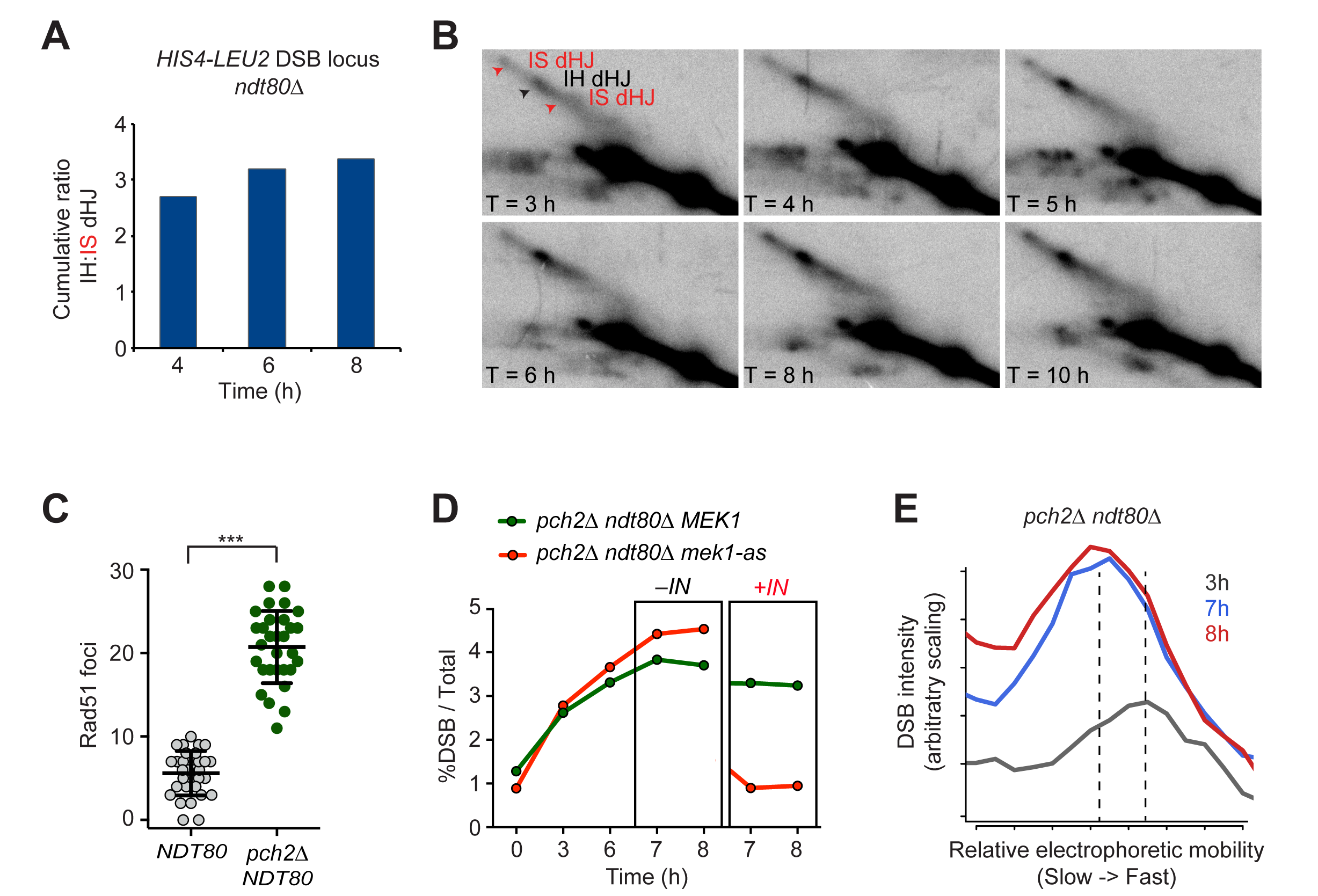

Supplement: S8 Fig — (A) Ratio of interhomologue to intersister (IH:IS) dHJs at the HIS4-LEU2 DSB locus over time in prophase-arrested ndt80Δ cells (H2640). (B) Two-dimensional gel electrophoresis to resolve interhomologue (IH) and intersister (IS) dHJ species at the GAT1 DSB locus at different time points in meiosis (H7036). (C) Quantification of the number of Rad51 foci per spread nucleus in NDT80 (H574) and pch2Δ NDT80 (H3084) at T = 3 h. Only samples with complete SC were examined. n = 30; error bars are S.D. from the mean; *** p < 0.001 Mann-Whitney test. (D) Percentage of DSB fragments over total DNA at the ERG1 locus for pch2Δ ndt80Δ (H6639) and pch2Δ ndt80Δ mek1-as (H8360) at the indicated time point and treatment. (E) Relative electrophoretic mobility of DSBs fragments at the ERG1 locus in pch2Δ ndt80Δ (H6639) mutants (shown in Fig 6F). Dashed lines highlight the position of the respective maxima. (TIF) [file pbio.1002369.s009.tif]
