# Supplementary material for: Chromosome Synapsis Alleviates Mek1-Dependent Suppression of Meiotic DNA Repair
Source: PLoS Biol. 2016 Feb 12;14(2):e1002369. doi: 10.1371/journal.pbio.1002369 (PMC4752329; doi:10.1371/journal.pbio.1002369)
Supplement: S1 Table — (DOCX) [file pbio.1002369.s010.docx]

**S1 Table.**

| Strain (#) | No spores | Monads^a^ | Dyads^a^ | Tetrads^a^ | N^b^ | Sporulation (%) |
| --- | --- | --- | --- | --- | --- | --- |
| WT ^c^ (H7838) | 18 | 5 | 5 | 172 | 200 | 91.0 |
| WT ^c^ (H7838)  + Rapamycin ^d^ | 12 | 2 | 16 | 170 | 200 | 94.0 |
| *RDH54-FRB* ^c^ (H7847) | 19 | 0 | 23 | 158 | 200 | 90.5 |
| *RDH54-FRB* ^c^ (H7847)  + Rapamycin ^d^ | 97 | 48 | 33 | 22 | 200 | 51.5 |
| *RAD54-FRB* ^c^ (H7796) | 23 | 3 | 13 | 161 | 200 | 88.5 |
| *RAD54-FRB* ^c^ (H7796)  + Rapamycin ^d^ | 49 | 36 | 42 | 73 | 200 | 75.5 |
| *SPO11-FRB* ^c^ (H7792) | 22 | 4 | 10 | 164 | 200 | 89.0 |
| *SPO11-FRB* ^c^ (H7792)  + Rapamycin ^d^ | 35 | 21 | 41 | 103 | 200 | 82.5 |
| *MER2-FRB* ^c^ (H7834) | 10 | 2 | 24 | 164 | 200 | 95.5 |
| *MER2-FRB* ^c^ (H7834)  + Rapamycin ^d^ | 65 | 30 | 36 | 69 | 200 | 67.5 |
| *ZIP1-FRB* ^c^ (H7812) | 15 | 4 | 15 | 166 | 200 | 92.5 |
| *ZIP1-FRB* ^c^ (H7812)  + Rapamycin ^d^ | 144 | 17 | 14 | 25 | 200 | 28.0 |
|  |  |  |  |  |  |  |
| WT (H119) | 33 | 2 | 6 | 159 | 200 | 83.5 |
| WT (H119 )  + Rapamycin ^d^ | 148 | 8 | 6 | 38 | 200 | 26.0 |
|  |  |  |  |  |  |  |
| *zip1Δ* ^c^ (H7794) | 180 | 6 | 5 | 9 | 200 | 10.0 |
| *spo11Δ* ^c^ (H7795) | 152 | 8 | 11 | 29 | 200 | 24.0 |
| *rad54Δ* ^c^ (H7813) | 72 | 13 | 38 | 77 | 200 | 64.0 |

^a^ Monad- single spore, dyad- two spores, tetrad- three/four spores

^b^ N - number of meiotic cells analyzed

^c^ *RPL13A-2xFKBP12::TRP1/RPL13A-2xFKBP12::TRP1 fpr1::KANMX4/fpr1::KANMX4 tor1-1::HIS3/tor1-1::HIS3* (anchor-away strain background)

^d^ Rapamycin was added to the cells when meiosis was induced (T=0 hr)
