# Supplementary material for: Chromosome Synapsis Alleviates Mek1-Dependent Suppression of Meiotic DNA Repair
Source: PLoS Biol. 2016 Feb 12;14(2):e1002369. doi: 10.1371/journal.pbio.1002369 (PMC4752329; doi:10.1371/journal.pbio.1002369)
Supplement: S2 Table — (DOCX) [file pbio.1002369.s011.docx]

**S2 Table.**

| Strain (#) | 4- viable | 3-viable | 2-viable | 1-viable | 0-viable | N ^a^ | Viability (%) |
| --- | --- | --- | --- | --- | --- | --- | --- |
| WT ^b^ (H7838) | 59 | 6 | 1 | 0 | 0 | 66 | 96.9 |
| WT ^b^ (H7838)  + Rapamycin ^c^ | 61 | 4 | 1 | 0 | 0 | 66 | 97.7 |
| *RDH54-FRB* ^b^ (H7847) | 63 | 0 | 3 | 0 | 0 | 66 | 97.7 |
| *RDH54-FRB* ^b^ (H7847)  + Rapamycin ^c^ | 55 | 8 | 2 | 0 | 1 | 66 | 93.9 |
| *RAD54-FRB* ^b^ (H7796) | 61 | 2 | 2 | 0 | 0 | 65 | 97.7 |
| *RAD54-FRB* ^b^ (H7796)  + Rapamycin ^c^ | 17 | 29 | 11 | 7 | 1 | 65 | 70.8 |
| *SPO11-FRB* ^b^ (H7792) | 49 | 12 | 5 | 0 | 0 | 66 | 91.7 |
| *SPO11-FRB* ^b^ (H7792)  + Rapamycin ^c^ | 6 | 1 | 0 | 0 | 59 | 66 | 10.2 |
| *MER2-FRB* ^b^ (H7834) | 86 | 3 | 8 | 0 | 2 | 99 | 93.2 |
| *MER2-FRB* ^b^ (H7834)  + Rapamycin ^c^ | 0 | 0 | 1 | 0 | 98 | 99 | 0.5 |
| *ZIP1-FRB* ^b^ (H7812) | 56 | 9 | 1 | 0 | 0 | 66 | 95.1 |
| *ZIP1-FRB* ^b^ (H7812)  + Rapamycin ^c^ | 23 | 13 | 11 | 2 | 17 | 66 | 58.7 |
|  |  |  |  |  |  |  |  |
| WT (H119) | 60 | 3 | 1 | 2 | 0 | 66 | 95.8 |
| WT (H119)  + Rapamycin ^c^ | 36 | 20 | 6 | 3 | 0 | 65 | 84.2 |
|  |  |  |  |  |  |  |  |
| *zip1Δ* ^b^ (H7794) | 24 | 5 | 10 | 2 | 25 | 66 | 50.4 |
| *spo11Δ* ^b^ (H7795) | 0 | 0 | 2 | 0 | 64 | 66 | 1.5 |
| *rad54Δ* ^b^ (H7813) | 3 | 22 | 22 | 12 | 7 | 66 | 50.8 |

^a^ N - number of tetrads analyzed

^b^ *RPL13A-2xFKBP12::TRP1/RPL13A-2xFKBP12::TRP1 fpr1::KANMX4/fpr1::KANMX4 tor1-1::HIS3/tor1-1::HIS3* (anchor-away strain background)

^c^ Rapamycin - rapamycin was added to the cells when meiosis was induced (T=0 hr)
