# Supplementary material for: Chromosome Synapsis Alleviates Mek1-Dependent Suppression of Meiotic DNA Repair
Source: PLoS Biol. 2016 Feb 12;14(2):e1002369. doi: 10.1371/journal.pbio.1002369 (PMC4752329; doi:10.1371/journal.pbio.1002369)
Supplement: S3 Table — (DOCX) [file pbio.1002369.s012.docx]

**S3 Table.**

| **Strain** | **Genotype** |
| --- | --- |
| H7121 | *MATa/MATα ho::LYS2/ ho::LYS2 lys2/lys2 URA3/URA3 leu2::hisG/LEU2 his3::hisG/his3::hisG trp1::hisG/trp1::hisG RPL13A-2xFKBP12::TRP1/RPL13A-2xFKBP12::TRP1 fpr1::KANMX4/fpr1::KANMX4 tor1-1::HIS3/tor1-1::HIS3 ndt80::TRP1/ndt80::TRP1 RAD54-FRB::KANMX6/RAD54-FRB::KANMX6* |
| H7485 | *MATa/MATα ho::LYS2/ ho::LYS2 lys2/lys2 ura3/URA3 leu2::hisG/LEU2 his3::hisG/his3::hisG trp1::hisG/trp1::hisG RPL13A-2xFKBP12::TRP1/RPL13A-2xFKBP12::TRP1 fpr1::KANMX4/fpr1::KANMX4 tor1-1::HIS3/tor1-1::HIS3 ndt80::TRP1/ndt80::TRP1 RDH54-FRB::KANMX6/RDH54-FRB::KANMX6* |
| H7137 | *MATa/MATα ho::LYS2/ ho::LYS2 lys2/lys2 URA3/ura3 LEU2/LEU2 his3::hisG/his3::hisG trp1::hisG/trp1::hisG RPL13A-2xFKBP12::TRP1/RPL13A-2xFKBP12::TRP1 fpr1::KANMX4/fpr1::KANMX4 tor1-1::HIS3/tor1-1::HIS3 ndt80::TRP1/ndt80::TRP1* |
| H7421 | *MATa/MATα ho::LYS2/ ho::LYS2 lys2/lys2 URA3/ura3 leu2::hisG/LEU2 his3::hisG/his3::hisG trp1::hisG/trp1::hisG RPL13A-2xFKBP12::TRP1/RPL13A-2xFKBP12::TRP1 fpr1::KANMX4/fpr1::KANMX4 tor1-1::HIS3/tor1-1::HIS3 ndt80::TRP1/ndt80::TRP1 zip1::ZIP1-FRB/zip1::ZIP1-FRB* |
| H7136 | *MATa/MATα ho::LYS2/ ho::LYS2 lys2/lys2 URA3/URA3 leu2/LEU2 his3::hisG/his3::hisG trp1::hisG/trp1::hisG RPL13A-2xFKBP12::TRP1/RPL13A-2xFKBP12::TRP1 fpr1::KANMX4/fpr1::KANMX4 tor1-1::HIS3/tor1-1::HIS3 ndt80::TRP1/ndt80::TRP1 PSY2-FRB::KANMX6/PSY2-FRB::KANMX6* |
| H7740 | *MATa/MATα ho::LYS2/ ho::LYS2 lys2/lys2 URA3/ura3 LEU2/LEU2 his3::hisG/his3::hisG trp1::hisG/trp1::hisG RPL13A-2xFKBP12::TRP1/RPL13A-2xFKBP12::TRP1 fpr1::KANMX4/fpr1::KANMX4 tor1-1::HIS3/tor1-1::HIS3 ndt80::TRP1/ndt80::TRP1 RAD54-FRB::KANMX6/RAD54-FRB::KANMX6 SPO11-FRB::KANMX6/SPO11-FRB::KANMX6* |
| H7840 | *MATa/MATα ho::LYS2/ ho::LYS2 lys2/lys2 ura3/URA3 LEU2/LEU2 his3::hisG/his3::hisG trp1::hisG/trp1::hisG RPL13A-2xFKBP12::TRP1/RPL13A-2xFKBP12::TRP1 fpr1::KANMX4/fpr1::KANMX4 tor1-1::HIS3/tor1-1::HIS3 ndt80::TRP1/ndt80::TRP1 RAD54-FRB::KANMX6/RAD54-FRB::KANMX6 MER2-FRB::KANMX6/MER2-FRB::KANMX6* |
| H7036 | *MATa/MATα ho::LYS2/ ho::LYS2* *leu2::hisG/leu2::hisG ura3/ura3::YIplac211:URA3 ndt80::LEU2/ndt80::LEU2 GAT1(KpnI-BamHI-KpnI)/GAT1(KpnI-x-KpnI)* |
| H6179 | *MATa/MATα ho::LYS2/ ho::LYS2 lys2/lys2 his4X/HIS4 ura3/URA3 leu2::hisG/LEU2 trp1::hisG/TRP1 ndt80::TRP1/ndt80::TRP1* |
| H7473 | *MATa/MATα ho::LYS2/ ho::LYS2 lys2/lys2 ura3/ura3 his3::hisG/HIS3 TRP1/TRP1 ndt80::TRP1/ndt80::TRP1 sae2::natmx4::SAE2-3HA::URA3::leu2/sae2::natmx4::SAE2-3HA::URA3::leu2* |
| H7413 | *MATa/MATα ho::LYS2/ ho::LYS2 lys2/lys2 his4X/HIS4 ura3/URA3 leu2::hisG/LEU2 trp1::hisG/trp1::hisG ndt80::TRP1/ndt80::TRP1 MEK1-GFP::KANMX6/MEK1-GFP::KANMX6* |
| H7561 | *MATa/MATα ho::LYS2/ ho::LYS2 lys2/lys2 trp1::hisG/trp1::hisG ndt80::TRP1/ndt80::TRP1 MEK1-GFP::KANMX6/MEK1-GFP::KANMX6 zip3::KANMX6/zip3::KANMX6* |
| H6731 | *MATa/MATα ho::LYS2/ ho::LYS2 lys2/lys2 HIS3/HIS3 ura3/ura3 LEU2/LEU2 TRP1/TRP1 YCR047c::caURA3MX6/YCR047c ndt80::TRP1/ndt80::TRP1* |
| H6732 | *MATa/MATα ho::LYS2/ ho::LYS2 lys2/lys2 his3/HIS3 ura3/URA3 leu2::hisG/LEU2 trp1::hisG?/trp1::hisG YCR047c::caURA3MX6/YCR047c arg4-Bgl/ARG4 ndt80::TRP1/ndt80::TRP1 zip1::LYS2/zip1::LYS2* |
| H3928 | *MATa/MATα ho::LYS2/ ho::LYS2 lys2/lys2 his4X/his4X ura3/ura3 leu2::hisG/leu2::hisG trp1::hisG/trp1::hisG ndt80::TRP1/ndt80::TRP1 pph3::LEU2/PPH3 mek1::KANMX4/MEK1* |
| H3929 | *MATa/MATα ho::LYS2/ ho::LYS2 lys2/lys2 his4X/HIS4 ura3/ura3 leu2::hisG/leu2::hisG trp1::hisG/trp1::hisG ndt80::TRP1/ndt80::TRP1 pph3::LEU2/pph3::LEU2 mek1::KANMX4/MEK1* |
| H6639 | *MATa/MATα ho::LYS2/ ho::LYS2 lys2/lys2 his4X/his4X his3::hisG/HIS3 ura3/URA3 leu2::hisG/LEU2 trp1::hisG?/TRP1 ndt80::TRP1/ndt80::TRP1 pch2::KANMX4/pch2::KANMX4* |
| H6704 | *MATa/MATα ho::LYS2/ ho::LYS2 lys2/lys2 leu2::hisG/LEU2 his3::hisG/HIS3 trp1::hisG?/trp1::hisG zip1::LYS2/zip1::LYS2 ura3::zip1-4LA::URA3/ura3::zip1-4LA::URA3* |
| H8124 | *MATa/MATα ho::LYS2/ ho::LYS2* *lys2/lys2 leu2::hisG/LEU2 HIS3/HIS3 trp1::hisG/TRP1 ura3/URA3 ndt80::LEU2/ndt80::LEU2 ZIP3-6HA::KANMX6/ZIP3* |
| H7495 | *MATa/MATα ho::LYS2/ ho::LYS2 lys2/lys2 ura3/ura3 leu2::hisG/LEU2 his3::hisG/His3 TRP1?/TRP1 ndt80::TRP1/ndt80::TRP1 REC8-3HA::URA3/REC8-3HA::URA3* |
| H7496 | *MATa/MATα ho::LYS2/ ho::LYS2 lys2/lys2 ura3/ura3 leu2::hisG/LEU2 his3::hisG/His3 TRP1?/TRP1 ndt80::TRP1/ndt80::TRP1 REC8-3HA::URA3/REC8-3HA::URA3 pch2::KANMX4/pch2::KANMX4* |
| H7562 | *MATa/MATα ho::LYS2/ ho::LYS2 lys2/lys2 leu2::hisG/LEU2 his3::hisG/His3 trp1::hisG/trp1::hisG ndt80::TRP1/ndt80::TRP1 REC8-3HA::URA3/REC8-3HA::URA3 ura3::zip1-4LA::URA3/ura3::zip1-4LA::URA3* |
| H7792 | *MATa/MATα ho::LYS2/ ho::LYS2 lys2/lys2 ura3/URA3 LEU2/LEU2 his3::hisG/his3::hisG trp1::hisG/trp1::hisG RPL13A-2xFKBP12::TRP1/RPL13A-2xFKBP12::TRP1 fpr1::KANMX4/fpr1::KANMX4 tor1-1::HIS3/tor1-1::HIS3 SPO11-FRB::KANMX6/SPO11-FRB::KANMX6* |
| H7847 | *MATa/MATα ho::LYS2/ ho::LYS2 lys2/lys2 URA3/URA3 leu2::hisG/LEU2 his3::hisG/his3::hisG trp1::hisG/trp1::hisG RPL13A-2xFKBP12::TRP1/RPL13A-2xFKBP12::TRP1 fpr1::KANMX4/fpr1::KANMX4 tor1-1::HIS3/tor1-1::HIS3 RDH54- FRB::KANMX6/RDH54-FRB::KANMX6* |
| H7796 | *MATa/MATα ho::LYS2/ ho::LYS2 lys2/lys2 ura3/URA3 leu2::hisG/LEU2 his3::hisG/his3::hisG trp1::hisG/trp1::hisG RPL13A-2xFKBP12::TRP1/RPL13A-2xFKBP12::TRP1 fpr1::KANMX4/fpr1::KANMX4 tor1-1::HIS3/tor1-1::HIS3 RAD54- FRB::KANMX6/RAD54-FRB::KANMX6* |
| H7834 | *MATa/MATα ho::LYS2/ ho::LYS2 lys2/lys2 URA3/URA3 leu2::hisG/LEU2 his3::hisG/his3::hisG trp1::hisG/trp1::hisG RPL13A-2xFKBP12::TRP1/RPL13A-2xFKBP12::TRP1 fpr1::KANMX4/fpr1::KANMX4 tor1-1::HIS3/tor1-1::HIS3 MER2- FRB::KANMX6/MER2-FRB::KANMX6* |
| H7838 | *MATa/MATα ho::LYS2/ ho::LYS2 lys2/lys2 ura3/URA3 leu2::hisG/LEU2 his3::hisG/his3::hisG trp1::hisG/trp1::hisG RPL13A-2xFKBP12::TRP1/RPL13A-2xFKBP12::TRP1 fpr1::KANMX4/fpr1::KANMX4 tor1-1::HIS3/tor1-1::HIS3* |
| H7812 | *MATa/MATα ho::LYS2/ ho::LYS2 lys2/lys2 URA3/URA3 leu2::hisG/LEU2 his3::hisG/his3::hisG trp1::hisG/trp1::hisG RPL13A-2xFKBP12::TRP1/RPL13A-2xFKBP12::TRP1 fpr1::KANMX4/fpr1::KANMX4 tor1-1::HIS3/tor1-1::HIS3 zip1::ZIP1-FRB/zip1::ZIP1-FRB* |
| H0119 | *MATa/MATα ho::LYS2/ ho::LYS2 lys2/lys2 his4B::LEU2/his4X::LEU2(Bam)::URA3 arg4-Nsp/arg4-BglII ura3/ura3 leu2::hisG/leu2::hisG* |
| H7839 | *MATa/MATα ho::LYS2/ ho::LYS2 lys2/lys2 URA3/URA3 leu2::hisG/LEU2 his3::hisG/his3::hisG trp1::hisG/trp1::hisG RPL13A-2xFKBP12::TRP1/RPL13A-2xFKBP12::TRP1 fpr1::KANMX4/fpr1::KANMX4 tor1-1::HIS3/tor1-1::HIS3 ndt80::TRP1/ndt80::TRP1 MER2-FRB::KANMX6/MER2-FRB::KANMX6* |
| H7793 | *MATa/MATα ho::LYS2/ ho::LYS2 lys2/lys2 ura3/URA3 leu2::hisG/LEU2 his3::hisG/his3::hisG trp1::hisG/trp1::hisG RPL13A-2xFKBP12::TRP1/RPL13A-2xFKBP12::TRP1 fpr1::KANMX4/fpr1::KANMX4 tor1-1::HIS3/tor1-1::HIS3 ndt80::TRP1/ndt80::TRP1 SPO11-FRB::KANMX6/SPO11-FRB::KANMX6* |
| H7794 | *MATa/MATα ho::LYS2/ ho::LYS2 lys2/lys2 ura3/ura3 leu2::hisG/LEU2 his3::hisG/his3::hisG trp1::hisG/trp1::hisG RPL13A-2xFKBP12::TRP1/RPL13A-2xFKBP12::TRP1 fpr1::KANMX4/fpr1::KANMX4 tor1-1::HIS3/tor1-1::HIS3 zip1::URA3/zip1::URA3* |
| H7795 | *MATa/MATα ho::LYS2/ ho::LYS2 lys2/lys2 ura3/ura3 leu2::hisG/LEU2 his3::hisG/his3::hisG trp1::hisG/trp1::hisG RPL13A-2xFKBP12::TRP1/RPL13A-2xFKBP12::TRP1 fpr1::KANMX4/fpr1::KANMX4 tor1-1::HIS3/tor1-1::HIS3 spo11::URA3/spo11::URA3* |
| H7799 | *MATa/MATα ho::LYS2/ ho::LYS2 lys2/lys2 URA3/URA3 leu2::hisG/LEU2 his3::hisG/his3::hisG trp1::hisG/trp1::hisG RPL13A-2xFKBP12::TRP1/RPL13A-2xFKBP12::TRP1 fpr1::KANMX4/fpr1::KANMX4 tor1-1::HIS3/tor1-1::HIS3 rdh54::KANMX4/rdh54::KANMX4* |
| H7813 | *MATa/MATα ho::LYS2/ ho::LYS2 lys2/lys2 ura3/ura3 leu2::hisG/LEU2 his3::hisG/his3::hisG trp1::hisG/trp1::hisG RPL13A-2xFKBP12::TRP1/RPL13A-2xFKBP12::TRP1 fpr1::KANMX4/fpr1::KANMX4 tor1-1::HIS3/tor1-1::HIS3 rad54::URA3/rad54::URA3* |
| H2640 | *MATa/MATα ho::LYS2/ ho::hisG? lys2/LYS2 leu2::hisG/leu2::hisG ura3(ΔSma-Pst)/ura3(ΔSma-Pst) his4X::LEU2-(NBam)-URA3/HIS4::LEU2-(NBam) ndt80::LEU2/ndt80::LEU2* |
| H8359 | *MATa/MATα ho::LYS2/ ho::LYS2 lys2/lys2 URA3/URA3 leu2::hisG/LEU2 his3::hisG/his3::hisG trp1::hisG/trp1::hisG RPL13A-2xFKBP12::TRP1/RPL13A-2xFKBP12::TRP1 fpr1::KANMX4/fpr1::KANMX4 tor1-1::HIS3/tor1-1::HIS3 zip1::ZIP1-FRB/zip1::ZIP1-FRB MER2-FRB::KANMX6/MER2-FRB::KANMX6 ndt80::TRP1/ndt80::TRP1* |
| H6639 | *MATa/MATα ho::LYS2/ ho::LYS2 lys2/lys2 URA3/ura3 leu2::hisG/ LEU2 his4X /HIS4 trp1::hisG/TRP1 ndt80::TRP1/ndt80::TRP1 pch2::KANMX4/pch2::KANMX4* |
| H8360 | *MATa/MATα ho::LYS2/ ho::LYS2 lys2/lys2 URA3/ura3 leu2::hisG/ leu2::hisG his3::hisG/his3::hisG trp1::hisG/trp1::hisG ndt80::TRP1/ndt80::TRP1 pch2::KANMX4/pch2::KANMX4 mek1::LEU2/mek1::LEU2 ade2-Bgl::mek1-as1::ADE2/ ade2-Bgl::mek1-as1::ADE2* |
| H8210 | *MATa/MATα ho::LYS2/ ho::LYS2 lys2/lys2 ura3/ura3 leu2::hisG/ leu2::hisG hop1::LEU2/hop1::LEU2 hop1-T318A::URA3/ hop1-T318A::URA3* |
| H3454 | *MATa/MATα ho::LYS2/ ho::LYS2 lys2/lys2 ura3/ura3 leu2::hisG/ leu2::hisG hop1::LEU2/hop1::LEU2* |
| H574 | *MATa/MATα ho::LYS2/ ho::LYS2 lys2/lys2 URA3/URA3 leu2::hisG/ LEU2 his4X /HIS4 TRP1/TRP1* |
| H3084 | *MATa/MATα ho::LYS2/ ho::LYS2 lys2/lys2 ura3/URA3 leu2::hisG/ LEU2 his4X /HIS4 trp1::hisG/TRP1 pch2::KANMX4/pch2::KANMX4* |
| K303 | *MATa/MATα leu2-3,112/ leu2-3,112 his4-260,519/ his4-260,519 lys2ΔNhe/ lys2ΔNhe thr1-4/ thr1-4 ade2-1/ ade2-1 ura3-1/ura3-1 trp1-289/trp1-289 zip1-4LA/zip1-4LA ECM11-MYC::KANMX4/ECM11 ndt80Δ::LEU2/ndt80Δ::LEU2* |
| AM2981 | *MATa/MATα leu2-3,112/ leu2-3,112 his4-260,519/ his4-260,519 lys2ΔNhe/ lys2ΔNhe thr1-4/ thr1-4 ade2-1/ ade2-1 ura3-1/ura3-1 trp1-289/trp1-289 pch2::TRP1/pch2::TRP1 ECM11-MYC::KANMX4/ECM11 ndt80::LEU2/ndt80::LEU2* |
